# Effects of different exercise interventions on motor function in patients at different post-stroke recovery phases: a systematic review and Bayesian network meta-analysis

**DOI:** 10.3389/fneur.2025.1678951

**Published:** 2025-12-08

**Authors:** Longwei Chen, Liang Xia, Minghui Du, Yueying Liu, Mengyue Guo, Zeyi Zhang, Ya Zhao, Xiue Shi

**Affiliations:** 1 Sports Center, Xi’an Jiaotong University, Xi’an, Shaanxi, China; 2 Rehabilitation Science Institute, Shanxi Provincial Rehabilitation Hospital, Xi’an, Shaanxi, China; 3 Suzhou University, Suzhou, Jiangsu, China

**Keywords:** stroke, exercise intervention, motor function recovery, Bayesian network meta-analysis, recovery phases, post-stroke rehabilitation, randomized controlled trial

## Abstract

**Background:**

Post-stroke motor dysfunction is common, with diverse intervention strategies; however, their efficacy across recovery phases remains unclear.

**Objective:**

To systematically review and compare the effects of different exercise interventions on motor function in patients at different post-stroke recovery phases.

**Methods:**

Web of Science, PubMed, Embase, and the Cochrane Library were searched from inception to October 2025. Randomized controlled trials (RCTs) in adults with stroke assessing exercise effects on motor function were included. A Bayesian Network Meta-analysis (NMA) was applied for measures with enough studies and comparisons. Risk of bias was assessed using the Cochrane tool.

**Results:**

A total of 35 RCTs involving 1,540 stroke patients. In the acute phase, aerobic exercise (AE) was potentially superior to conventional therapy (CT) in improving 6-min walk distance (6MWD), Barthel Index (BI), and Fugl-Meyer Assessment (FMA). In the subacute phase, AE also showed a certain advantage in enhancing 6MWD. According to the surface under the cumulative ranking curve (SUCRA), core stability exercise combined with resistance exercise (CSE + RE, 99.99%) ranked best for improving the 10-meter walk test (10MWT); CSE (80.84%) was most effective for BI; and AE (78.73%) was optimal for improving FMA. In the chronic phase, AE + RE ranked first for improving 6MWD (83.33%) and FMA (99.90%); CSE (98.62%) was the most effective for 10MWT; and AE (87.85%) remained the best intervention for enhancing BI.

**Conclusion:**

Exercise interventions exert phase-specific effects on motor recovery post-stroke. These findings underscore the importance of tailored, phase-specific rehabilitation programs. Long-term efficacy and individualized optimization require further investigation.

**Systematic review registration:**

PROSPERO CRD42024607395.

## 1 Introduction

Stroke is one of the leading causes of mortality and disability worldwide (1). According to global statistics, approximately 12.2 million new stroke cases and 6.55 million stroke-related deaths occurred in 2019, resulting in an estimated 143 million disability-adjusted life years (DALYs) lost (2). Stroke not only poses a significant threat to patients’ health but also imposes a substantial burden on families and society as a whole (3). Among post-stroke disabilities, motor dysfunction is a primary concern, manifesting as impaired motor abilities, abnormal gait patterns, reduced endurance, and limitations in daily activities (4, 5). Consequently, effective strategies to facilitate motor function recovery in stroke patients have become a central focus of rehabilitation medicine research.

The importance of exercise-based interventions—including aerobic exercises (AE), resistance exercises (RE), core stability exercises (CSE), psychomotor exercises (PME), and multimodal exercise (ME)—in stroke rehabilitation has been extensively recognized (6). Studies have demonstrated that these interventions can promote motor function recovery through multiple mechanisms, including enhancing neuroplasticity (7, 8), increasing muscle strength and functional capacity (9, 10), improving cardiorespiratory adaptations and exercise endurance (11, 12), and facilitating sensorimotor integration and balance control (13). However, several limitations persist in the current body of research.

First, due to differences in the physiological mechanisms of various exercise interventions, each approach possesses unique rehabilitation benefits (14, 15). However, the majority of studies have focused on single-modality interventions, lacking systematic comparisons of the relative advantages and potential synergistic effects among different strategies (14, 16). Second, stroke rehabilitation is a dynamic, phase-dependent process, with substantial variations in neurological remodeling patterns, muscle adaptations, and rehabilitation needs across different recovery phases (17). Yet, most existing studies have primarily targeted specific phases (e.g., acute or chronic), with relatively little consideration for the adaptation of exercise strategies across the full spectrum of stroke recovery (18). Thus, in clinical practice, a major challenge remains: how to precisely tailor exercise interventions to the patient’s specific phase of recovery in order to optimize individualized rehabilitation programs.

This study utilizes network meta-analysis to systematically compare the effects of different exercise interventions on motor function across various stroke recovery phases. By identifying the optimal applicability of different exercise modalities at each phase of recovery, this study aims to provide a scientific foundation for phase-specific, personalized rehabilitation strategies. Ultimately, these findings may contribute to improved rehabilitation outcomes and enhanced quality of life for stroke patients.

## 2 Methods

This study followed the guidelines outlined in the Preferred Reporting Items for Systematic Reviews and Meta-Analyses (PRISMA) statement (19) and incorporates the PRISMA extension to ensure comprehensive reporting of methods and results (20). The study protocol has been registered in the International Prospective Register of Systematic Reviews (PROSPERO) (CRD42024607395) on November 14, 2024.

### 2.1 Search strategy

Independent searches were conducted by two investigators across Web of Science, PubMed, Embase, and Cochrane Library, covering the period from the inception of the databases to October 2025. Boolean logic operators were employed to link the subject terms with free-text terms, including “stroke,” “apoplexy,” “apoplectic stroke,” “cerebrovascular accident,” “brain accident,” “cerebrovascular injury,” “exercise,” “fitness training,” “physical activity,” “resistance exercise,” “aerobic exercise,” “high-intensity interval training,” “random,” “randomized controlled trial,” and “RCT.” The specific retrieval strategy can be found in Supplementary Table S1. Additionally, the reference lists of pertinent meta-analyses and reviews were examined systematically to reduce the likelihood of missing any studies that met the inclusion criteria.

### 2.2 Inclusion and exclusion criteria

The inclusion criteria were strictly based on the PICOS (Population, Intervention, Comparison, Outcome, and Study Design) principle (21). The specific inclusion criteria were as follows: (1) Population: Individuals in the acute, subacute, or chronic stages of stroke recovery (aged ≥18 years) were included, with no restrictions on post-stroke type (e.g., ischemic, hemorrhagic, transient ischemic attack and confirmed by CT or MRI), additionally, recurrent strokes are included. (2) Intervention: The intervention group received one or a combination of the following exercise interventions: aerobic exercise (AE), core stability exercise (CSE), psychomotor exercise (PME), resistance exercise (RE), and multimodal exercise (ME), as defined in Table 1. (3) Comparison: The control group received either conventional therapy (CT) or any of the aforementioned interventions. (4) Study Design: Only randomized controlled trials (RCTs) were included. (5) Outcome measures: At least one of the following outcome measures must have been reported: exercise endurance (6-Minute Walk Distance, 6MWD) (22), walking ability (10-Meter Walk Test, 10MWT) (23), activities of daily living (Barthel Index, BI) (24), or limb motor control (Fugl-Meyer Assessment, FMA) (25). Furthermore, during the data extraction phase, we identified that only a very limited number of studies reported peak oxygen uptake as an outcome measure. Consequently, this outcome was not included in our meta-analysis. (6) Recovery phases: The study must explicitly report the duration of stroke in the participants, categorized into acute phase (0–7 days), subacute phase (7 days–6 months), or chronic phase (≥6 months) (26). (7) Language restriction: Only studies published in English were included.

**Table 1:** Classification criteria for different types of exercise interventions.

Type	Definition
AE	Aerobic exercise, characterized by high repetitions and low resistance during skeletal muscle contraction, is a widely recognized method for enhancing aerobic capacity and promoting overall health (97). It plays a crucial role in maintaining homeostasis by regulating energy production rates, blood flow, and substrate utilization during physical activity (98)
CSE	Core stability exercise consists of a series of training methods aimed at enhancing the strength and endurance of core muscles to improve body stability and movement performance (99). By targeting core muscle groups, including the abdominal, back, and gluteal muscles, this training improves postural stability against gravitational forces (100)
PME	Psychomotor exercise includes training methods that integrate physical and cognitive interactions, aiming to improve overall functionality by combining movement with cognitive tasks (101). Examples include Tai Chi and yoga, which engage both physical and psychological resources to enhance cognitive performance and mental health (102)
RE	Resistance exercise includes strength training and self-administered weight-bearing exercises. Resistance can be applied using resistance bands, body weight, or external weights such as dumbbells, barbells, machines, and kettlebells. When using external weights, the load is typically determined based on the maximum number of repetitions an individual can perform (6)
ME	Multimodal exercise integrates various exercise modalities, such as aerobic training, strength training, balance training, and flexibility training, into a comprehensive program aimed at improving overall health, physical fitness, and cognitive function (103). This training approach combines different movement patterns to enhance physical strength, endurance, flexibility, and coordination (104)

The exclusion criteria were as follows: (1) Studies that did not specify the type of exercise intervention. (2) Conference papers, reviews, and non-RCT studies (e.g., case reports, observational studies, cross-sectional studies, and studies without a control group). (3) Studies with a high dropout rate. (4) Studies that could not be accessed or downloaded. (5) Studies with incomplete outcome data, in which the authors did not respond after three attempts to contact them. (6) Mixed diagnoses (e.g., stroke combined with traumatic brain injury) were excluded.

### 2.3 Study selection process

Studies were selected independently by two investigators. They independently employed Endnote X9 software for the screening process. Initially, duplicates were removed. Subsequently, documents such as conference papers, abstracts, and letters were excluded. Systematic reviews and reviews were then organized separately. Finally, the titles, abstracts, and full texts were read in sequence to determine the studies to be included. If any important information was missing from a study, the corresponding authors would be contacted via email or other means to ensure the completeness of the data.

### 2.4 Date extraction and quality assessment

Two investigators (LX and MHD) independently reviewed all articles and extracted data. The extracted content included basic information of each study (first author, publication year, and country), participant characteristics (post-stroke recovery phase, age, and sample size), intervention characteristics (interventions and duration), and outcome measures (6MWD, 10MWT, BI, and FMA). In the event of discrepancies during the data extraction, a third investigator (LWC) would join the discussion and assist in making the decision. Two investigators (LX and MHD) applied the second version of the risk of bias tool (ROB2) to evaluate the included articles in the following domains: (1) bias arising from the randomization process; (2) bias due to deviations from the intended interventions; (3) bias from missing outcome data; (4) bias in outcome measurement; (5) selective reporting bias in result (27). The risk of bias (ROB) was classified into three categories: low ROB, high ROB, and unclear. The evaluation process was conducted independently by two investigators. In the event of any disputes arising during the assessment, a third investigator (LWC) would be consulted to reach a consensus.

### 2.5 Data processing

Based on the post-stroke recovery phases in the included studies, we categorized the studies into the acute phase (0–7 days), subacute phase (7 days–6 months), and chronic phase (≥6 months) for systematic review and Bayesian network meta-analysis. Given that certain outcome measures in the acute and subacute phases did not meet the minimum sample size requirement for Bayesian network meta-analysis, this study adopted a qualitative evidence synthesis approach (28). The intervention effects were assessed by analyzing the magnitude of changes in the mean difference (MD) before and after the intervention, where a larger absolute increase in the MD value indicated a more pronounced intervention effect. Additionally, data dispersion was evaluated using the standard deviation (SD), where a smaller SD indicated higher result stability. The statistical significance of the differences was determined by referring to the *p*-values reported in the original studies.

For the other outcome measures mean difference (MD) was employed as the effect size, accompanied by a corresponding 95% credible interval (CI). According to the methods outlined in section 16.1.3.2 of the Cochrane Handbook version 5.0.2, the mean change and standard deviation before and after treatment were calculated. The extracted data were then analyzed using R version 4.4.1 and Stata version 15.0 software. Given the heterogeneity among the trials, a Bayesian hierarchical random-effects model was first used to analyze the multiple comparisons of different exercise interventions in stroke treatment (29, 30). All calculations and graphics were performed utilizing R 4.4.1 and Stata 15.1 software. In the network plot, the nodes represent different interventions, with the size of each node indicating the sample size. The edges connecting the nodes denote direct comparisons between interventions, where thicker edges indicate a greater number of corresponding studies. Based on the theory of likelihood function and certain prior assumptions, Bayesian inference was conducted using R version 4.4.1 software. The Markov Chain Monte Carlo simulation was employed. A total of 500,000 iterations were set, with an annealing phase comprising 20,000 iterations, to investigate the posterior distribution of the analyzed nodes (31–33). The overall consistency of the results was assessed by comparing the difference in the values of deviance information criterion (DIC) between the consistency model and the inconsistency model. A DIC difference of less than 5 indicated favorable consistency. The presence of statistical heterogeneity within the entire network was assessed using the I^2^ statistic, which describes the percentage of total variation across studies that is due to heterogeneity rather than chance. For the interpretation of I^2^, values of 25, 50, and 75% were considered to represent low, moderate, and high heterogeneity, respectively. The node-splitting technique was applied to evaluate the local inconsistency in results with closed loops. The connections between various treatment methods were illustrated using a network plot. Simultaneously, a corrected funnel plot was employed to assess the risk of potential publication bias (34, 35). In addition, the exercise interventions studied were ranked utilizing the surface under the cumulative ranking curve (SUCRA). The SUCRA values ranged from 0 to 1. A higher SUCRA value indicated a higher ranking of an exercise intervention in improving specific indicators in stroke patients (36, 37). Ultimately, a league table was generated to display the results of pairwise comparisons between each of the interventions.

### 2.6 Meta-regression analysis

To explore the influence of potential effect modifiers on the intervention outcomes, we performed meta-regression analyses. These analyses were conducted to examine whether patient age and intervention duration significantly moderate the treatment effects of various exercise interventions compared to conventional therapy (CT). Separate regression models were developed for each of the four primary outcome measures: 6MWD, 10MWT, BI, and FMA. Using comparison type (e.g., AE vs. CT) as the unit of analysis, we incorporated age (defined as the mean age of the study sample) and intervention duration (in weeks) as continuous covariates into a Bayesian random-effects model. The results are presented as regression coefficients (β) along with their 95% credible intervals (CrI). The β coefficient represents the change in the mean difference (MD) of the outcome measure associated with a one-unit increase in age or intervention duration. Additionally, we reported the I^2^ statistic for the model residuals to quantify the proportion of residual heterogeneity not explained by the covariates.

### 2.7 Assessment of the quality of evidence

To evaluate the quality of evidence for the results of this network meta-analysis, we applied the GRADE approach using the CINeMA framework for the four primary outcomes: the 6-Minute Walk Test, the 10-Meter Walk Test, the Barthel Index, and the Fugl-Meyer Assessment. This framework evaluates evidence across six domains: risk of bias, publication bias, indirectness, imprecision, heterogeneity, and incoherence. Based on a comprehensive evaluation of these domains for each comparison, the quality of evidence was categorized into one of four grades: high, moderate, low, or very low.

## 3 Results

### 3.1 Retrieval results

**Figure 1:**
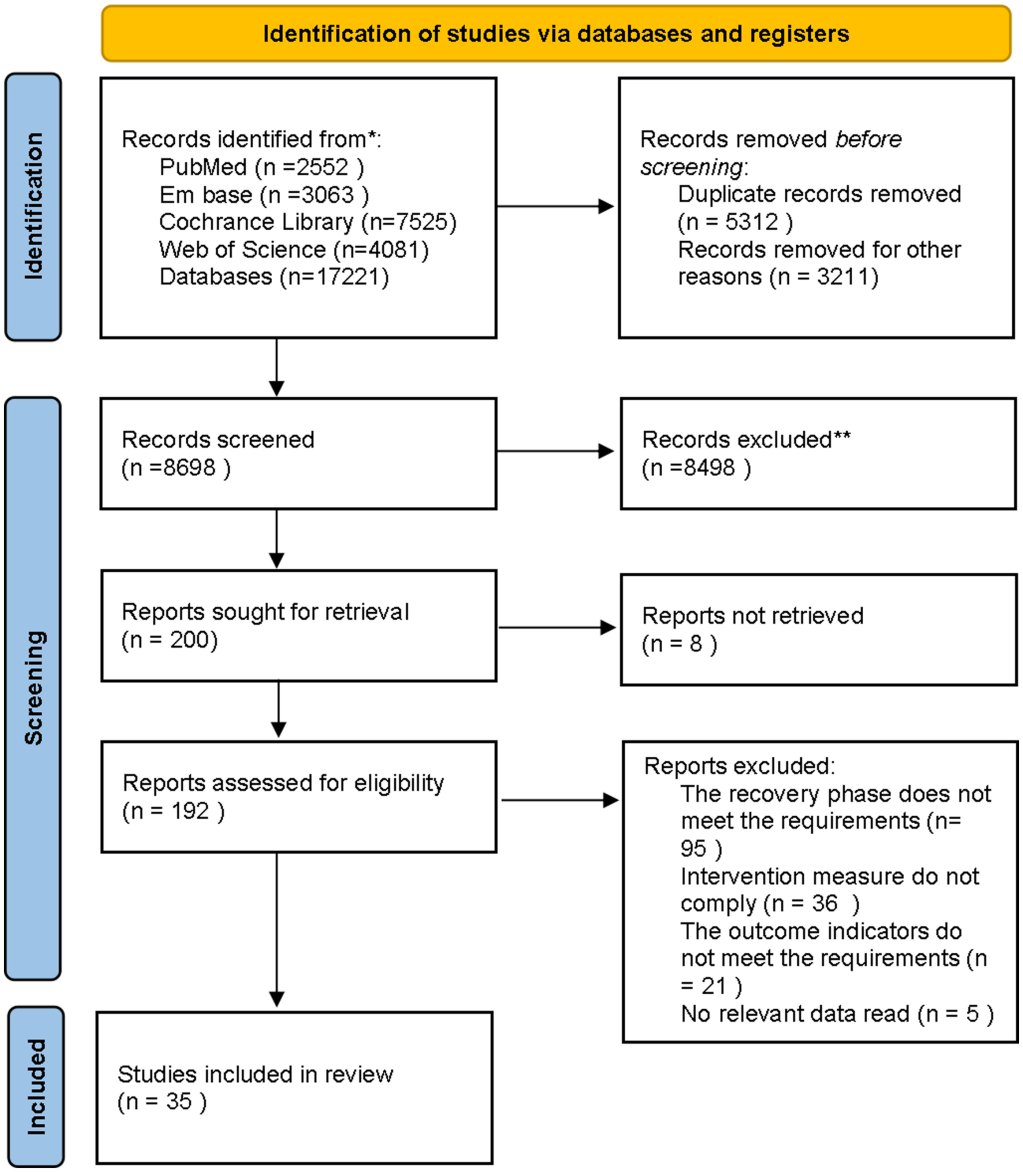
Literature screening flowchart.

Through a systematic search, we initially identified 17,221 records. After removing duplicates, 11,909 unique records remained for further screening. Subsequently, we excluded non-English publications, reviews, systematic reviews, conference abstracts, letters, retracted studies, short reports, and other irrelevant literature, narrowing the selection to 8,698 studies for title and abstract screening. After a rigorous evaluation, 192 studies met the predefined inclusion criteria. Finally, 35 studies were selected for inclusion following a full-text review (Figure 1) (38–72).

### 3.2 Characteristics of the included studies

Thirty-five randomized controlled trials (RCTs) (38–72) with a total of 1,540 patients were finally included. The main characteristics of the included studies are detailed in Table 2. According to the recovery phases of post-stroke patients: 2 studies (38, 39) in the acute phase, 9 studies (40–48) in the subacute phase, and 24 studies (49–72) in the chronic phase. Eight types of interventions were involved: 18 studies used AE (38, 39, 42–45, 47–49, 51–53, 56, 59, 67–69, 71), five studies used CSE (41, 46, 54, 55, 61), five studies used and PME (40, 49, 50, 63, 72), and four studies used RE (57, 58, 64, 70), 3 studies used ME (62, 65, 66), 4 studies used AE + RE (43, 59, 60, 67), and 1 study used CSE + RE (41). Primary outcomes included 6MWD in 15 studies (38, 43, 45, 49, 52–54, 58–60, 62, 64, 68–70), 10MWT in 9 studies (41, 45, 47, 54–56, 62, 63, 72), 10 studies (38, 39, 42–44, 46, 50, 51, 65, 66) using BI, and 9 studies (39, 40, 42, 44, 48, 51, 57, 67, 71) using FMA.

**Table 2:** Basic information of the included studies.

Study ID	Country	Sample	Age (M ± SD)	Time post-stroke [M ± SD/SE; M(IQR)]	Type of intervention	Intervention duration (week)	Outcomes
Saadatnia et al. (38)	Sweden	T1: 29 T2: 23	T1: 76.30 ± 6.40 T2: 72.10 ± 11.70	T1: 2.6 ± 1.8 (d) T2: 1.9 ± 1.0 (d)	T1: Conventional therapy T2: Aerobic exercise	3 weeks	6-Minute Walk Distance; Barthel Index
Sandberg et al. (39)	Iran	T1: 20 T2: 20	T1: 62 ± 12.40 T2: 66 ± 10.30		T1: Conventional therapy T2: Aerobic exercise	12 weeks	Fugl-Meyer Assessment; Barthel Index
Zhang et al. (40)	China	T1: 80 T2: 80	T1: 62.80 ± 11.18 T2: 65.44 ± 9.16	T1: 76.88 ± 61.63 (d) T2: 67.04 ± 47.86 (d)	T1: Conventional therapy T2: Psychomotor exercise	2 weeks	Fugl-Meyer Assessment
Kim et al. (41)	Korea	T1: 10 T2: 10 T3: 10	T1: 61.60 ± 3.92 T2: 61.50 ± 8.04 T3: 61.70 ± 6.66	T1: 2.75 ± 1.40 (m) T2: 2.62 ± 1.30 (m) T3: 2.70 ± 1.36 (m)	T1: Conventional therapy T2: Core stability exercise _ Resistance exercise T3: Core stability exercise	8 weeks	10-Meter Walk Test
Chen et al. (42)	China	T1: 62 T2: 59	T1: 56.41 ± 6.13 T2: 55.41 ± 6.78	T1: 3.23 ± 0.82(m) T2: 3.41 ± 0.79(m)	T1: Conventional therapy T2: Aerobic exercise	48 weeks	Fugl-Meyer Assessment; Barthel Index
Nave et al. (43)	Germany	T1: 85 T2: 87	T1: 70.00 ± 11.00 T2: 69.00 ± 12.00	T1: 27(17–41) (d) T2: 30(17–39) (d)	T1: Conventional therapy T2: Aerobic exercise	4 weeks	6-Minute Walk Distance; Barthel Index
Lee et al. (44)	Korea	T1: 18 T2: 19	T1: 63.67 ± 11.37 T2: 57.58 ± 13.98	T1: 29.22 ± 19.94 (d) T2: 30.37 ± 21.92 (d)	T1: Aerobic exercise T2: Aerobic exercise + Resistance exercise	4 weeks	Fugl-Meyer Assessment; Barthel Index
Sandberg et al. (45)	Sweden	T1: 27 T2: 29	T1:70.40 ± 8.10 T2: 71.30 ± 7.00	T1: 22.8 ± 10.8 (d) T2: 22.2 ± 10.1 (d)	T1: Conventional therapy T2: Aerobic exercise	12 weeks	6-Minute Walk Distance; 10-Meter Walk Test
Cabanas-Valdés et al. (46)	Spain	T1: 39 T2: 40	T1: 75.69 ± 9.40 T2: 74.92 ± 10.7	T1: 21.37 ± 16.00 (d) T2: 25.12 ± 17.30 (d)	T1: Conventional therapy T2: Core stability exercise	5 weeks	Barthel Index
Peurala et al. (47)	Finland	T1: 20	T1: 65.3 ± 9.90	T1: 7.8 ± 3.0 (d)	T1: Aerobic exercise	3 weeks	10-Meter Walk Test
Katz-Leurer et al. (48)	Israel	T1: 14 T2: 10	T1: 65 ± 9.00 T2: 59 ± 8.00		T1: Conventional therapy T2: Aerobic exercise	3 weeks	Fugl-Meyer Assessment
Caron et al. (49)	France	T1: 18 T2: 18	T1: 68.2 ± 10.10 T2: 67.1 ± 12.30	T1: 58.0(12.8–71.8) (m) T2: 45.0(15–73) (m)	T1: Aerobic exercise T2: Psychomotor exercise	12 weeks	6-Minute Walk Distance
Choi (50)	Korea	T1: 15 T2: 15	T1: 64.7 ± 14.30 T2: 62.50 ± 11.30	T1: 18.3 ± 7.0 (m) T2: 19.9 ± 7.1 (m)	T1: Conventional therapy T2: Psychomotor exercise	4 weeks	Barthel Index
Huang et al. (51)	China	T1: 12 T2: 12	T1: 63.33 ± 13.31 T2: 53.67 ± 9.16	T1: 33.75 ± 16.32 (m) T2: 28.67 ± 21.75 (m)	T1: Conventional therapy T2: Aerobic exercise	12 weeks	Fugl-Meyer Assessment; Barthel Index
Martins et al. (52)	Russia	T1: 18 T2: 18	T1: 55.00 ± 13.00 T2: 56.00 ± 17.00	T1: 41 ± 39 (m) T2: 52 ± 64 (m)	T1: Conventional therapy T2: Aerobic exercise	12 weeks	6-Minute Walk Distance
Lund et al. (53)	Denmark	T1: 13	T1: 67.70 ± 9.40	T1:16.8 ± 5.4 (m)	T1: Aerobic exercise	12 weeks	6-Minute Walk Distance
Lee et al. (54)	Korea	T1: 10 T2: 10	T1: 60.20 ± 8.24 T2: 59.80 ± 6.92	T1: 17.90 ± 3.51 (m) T2: 26.20 ± 1.99 (m)	T1: Conventional therapy T2: Core stability exercise	6 weeks	6-Minute Walk Distance; 10-Meter Walk Test
Lee and Han (55)	Korea	T1: 11 T2: 11	T1: 68.40 ± 3.70 T2: 69.20 ± 4.60		T1: Conventional therapy T2: Core stability exercise	4 weeks	10-Meter Walk Test
Kim and Lim (56)	Korea	T1: 6 T2: 7	T1: 64.50 ± 13.03 T2: 59.57 ± 11.75	T1: 29.16 ± 34.86 (m) T2: 33.85 ± 29.61 (m)	T1: Conventional therapy T2: Aerobic exercise	4 weeks	10-Meter Walk Test
Ellis et al. (57)	America	T1: 15 T2: 17	T1: 56.20 ± 12.90 T2: 59.80 ± 15.60	T1: 11.1 ± 6.1 (y) T2: 10.9 ± 6.5 (y)	T1: Conventional therapy T2: Resistance exercise	8 weeks	Fugl-Meyer Assessment
Vahlberg et al. (58)	Sweden	T1: 33 T2: 34	T1: 73.70 ± 5.30 T2: 72.60 ± 5.50	T1: 13 (2) (m) T2: 13 (4) (m)	T1: Conventional therapy T2: Resistance exercise	3 months	6-Minute Walk Distance
Lamberti et al. (59)	Italy	T1: 17 T2: 18	T1: 67.00 ± 10.00 T2: 69.00 ± 9.00	T1: 40 ± 51 (m) T2: 34 ± 46 (m)	T1: Aerobic exercise T2: Aerobic exercise + Resistance exercise	24 weeks	6-Minute Walk Distance
Kim et al. (60)	Korea	T1: 15 T2: 15	T1: 50.73 ± 13.50 T2: 48.27 ± 16.05	T1: 11.27 ± 4.10 (m) T2: 10.93 ± 3.67 (m)	T1: Conventional therapy T2: Aerobic exercise + Resistance exercise	4 weeks	6-Minute Walk Distance
Kılınç et al. (61)	Turkey	T1: 9 T2: 10	T1: 54.00 + 13.64 T2: 55.91 + 7.92	T1: 67.20 + 43.17 (m) T2: 58.66 + 55.68 (m)	T1: Conventional therapy T2: Core stability exercise	12 weeks	10-Meter Walk Test
Moore et al. (62)	Britain	T1: 20 T2: 20	T1: 70.00 ± 11.00 T2: 68.00 ± 8.00	T1: 16 ± 12 (m) T2: 21 ± 34 (m)	T1: Conventional therapy T2: Multimodal exercise	19 weeks	6-Minute Walk Distance
Kim et al. (63)	Korea	T1: 11 T2: 11	T1: 55.18 ± 10.20 T2: 53.45 ± 11.54		T1: Conventional therapy T2: Psychomotor exercise	6 weeks	10-Meter Walk Test
Kim et al. (64)	Korea	T1: 20 T2: 20	T1: 56.90 ± 4.30 T2: 57.30 ± 5.10	T1: 22 ± 5.4 (m) T2: 21 ± 5.1 (m)	T1: Conventional therapy T2: Resistance exercise	2 weeks	6-Minute Walk Distance
Kim et al. (65)	Norway	T1: 31 T2: 32	T1: 77.70 ± 8.90 T2: 72.30 ± 14.20		T1: Conventional therapy T2: Multimodal exercise	48 weeks	Barthel Index
Langhammer et al. (66)	Korea	T1: 14	T1: 58.20 ± 10.30	T1: 11.4 ± 2.7 (y)	T1: Multimodal exercise	6 weeks	Barthel Index
Page et al. (67)	America	T1: 4 T2: 3			T1: Aerobic exercise + Resistance exercise T2: Aerobic exercise	8 weeks	Fugl-Meyer Assessment
Sullivan et al. (68)	America	T1: 19	T1: 60.6 ± 13.7	T1: 27.5 ± 16.1 (m)	T1: Aerobic exercise	6 weeks	6-Minute Walk Distance
Pang et al. (69)	Canada	T1: 31 T2: 32	T1: 64.7 ± 8.4 T2: 65.8 ± 9.1	T1: 5.1 ± 3.6 (y) T2: 5.2 ± 5.0 (y)	T1: Conventional therapy T2: Aerobic exercise	19 weeks	6-Minute Walk Distance
Ouellette et al. (70)	America	T1: 21 T2: 21	T1: 66.1 ± 2.1 T2: 65.8 ± 2.5	T1: 25.6 ± 4.0 (SE, m) T2: 31.8 ± 3.3 (SE, m)	T1: Conventional therapy T2: Resistance exercise	12 weeks	6-Minute Walk Distance
Potempa et al. (71)	America	T1: 23 T2: 19			T1: Conventional therapy T2: Aerobic exercise	10 weeks	Fugl-Meyer Assessment
Promkeaw et al. (72)	Thailand	T1: 10 T2: 10	T1: 59.70 ± 10.70 T2: 62.50 ± 8.98	T1: 40.20 ± 4.98 (m) T2: 40.10 ± 6.38 (m)	T1: Conventional therapy T2: PME	3 weeks	10MWT

T1, T2, and T3 represent the different groups involved in a study.

### 3.3 Quality evaluation

**Figure 2:**
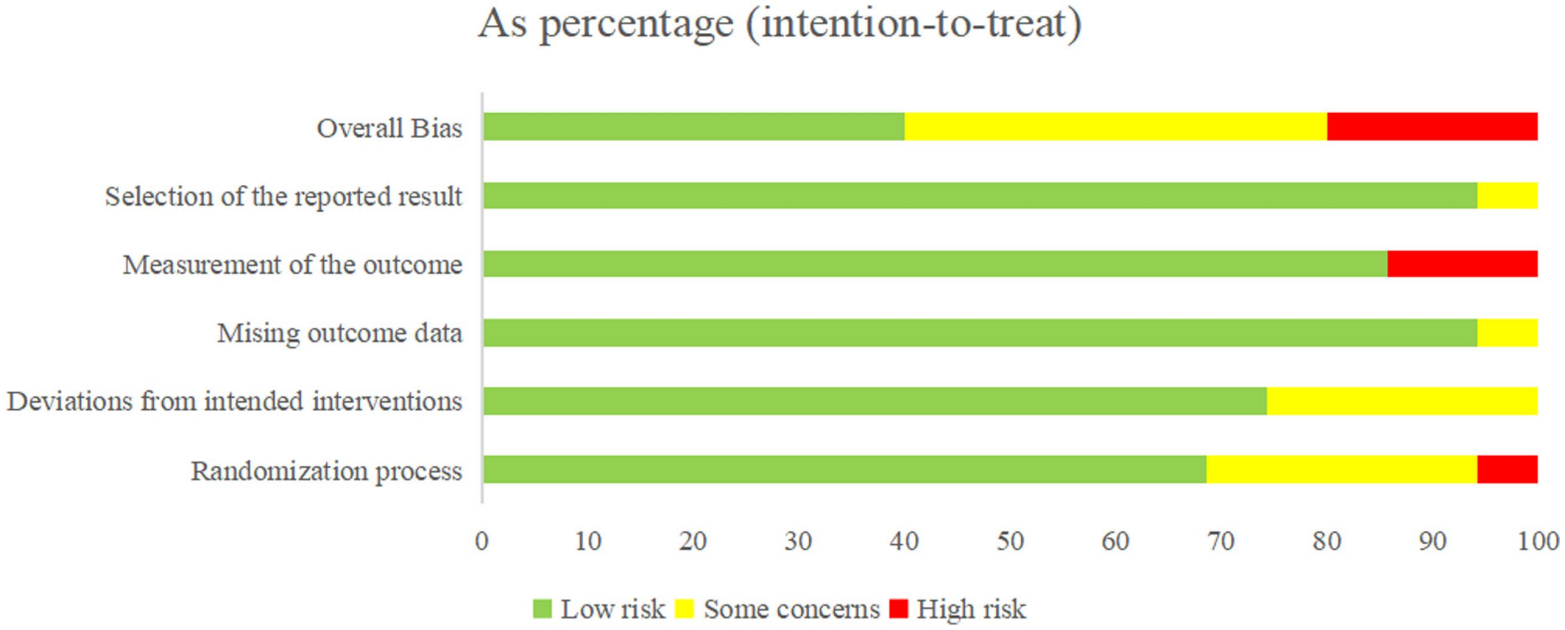
Overall quality evaluation results.

All included studies were randomized controlled trials (RCTs). Approximately 80% of the studies were categorized as low or uncertain risk of bias (ROB) in the overall assessment, with only a minority rated as high ROB (Figure 2).

**Figure 3:**
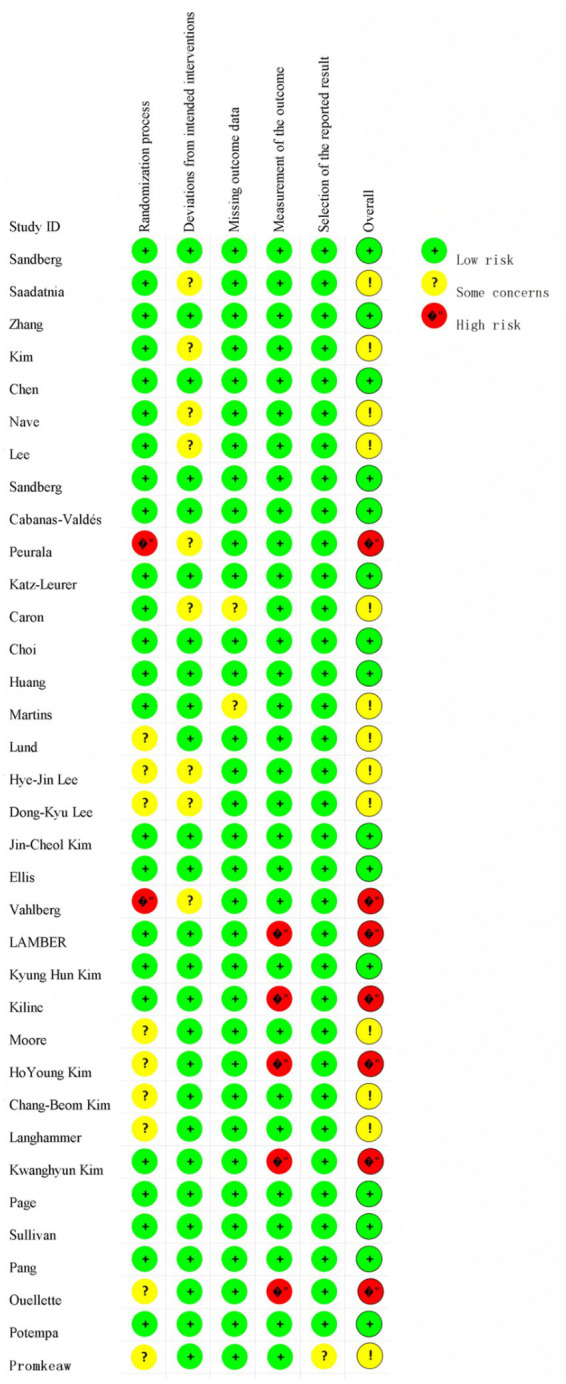
Quality evaluation of each study.

During the randomization process, 24 studies (38–46, 48–52, 56, 57, 59–61, 66–69, 71) had low ROB, 9 studies (53–55, 62–65, 70, 72) had an uncertain ROB due to unclear descriptions of randomization, and 2 studies (47, 58) did not report allocation concealment and were therefore categorized as high ROB. Regarding intervention deviation, 26 studies (38, 40, 42, 45, 46, 48, 50–53, 56, 57, 59–72) had low ROB, 9 studies (39, 41, 43, 44, 47, 49, 54, 55, 58) had an uncertain ROB due to environmental factors causing subject deviation from interventions. In terms of missing outcome data, 32 studies (38–48, 50, 51, 63–72) had low ROB, and 2 studies (49, 52) had an uncertain ROB due to less than 90% of the study population at the outcome. For outcome measures, 30 studies (38–58, 60, 62, 64, 65, 67–69, 71, 72) had low ROB, and 5 studies (59, 61, 63, 66, 70) had ROB due to the knowledge of the intervention by the person who measured it. Of the 35 included studies, 34 were rated as low risk of bias for selective reporting, and only one study was judged to have an unclear risk of bias (Figure 3).

### 3.4 Acute phase

#### 3.4.1 6MWD

One study (39) reported on 6MWD, including 52 patients. The study evaluated two interventions: CT and AE. The AE group showed greater improvement in 6MWD (MD = 22, SD = 200) compared to the CT group (MD = 0, SD = 152.59). Non-parametric tests in the original study (using MD and IQR) indicated that this difference was not statistically significant (*p* = 0.292).

#### 3.4.2 BI

Two studies (38, 39) reported on BI, including 92 patients. Both studies evaluated two interventions: CT and AE. In Study 1 (38), the AE group exhibited greater improvement in BI (MD = 6, SD = 8.89) compared to the CT group (MD = 3, SD = 7.41). Non-parametric tests in the original study (using MD and IQR) indicated that this difference was not statistically significant (*p* = 0.116). In Study 2 (39), the AE group also demonstrated a superior improvement in BI (MD = 70.5, SD = 2.34) compared to the CT group (MD = 4.4, SD = 2.48). The statistical analysis showed a significant difference in the AE group (*p* < 0.001), no statistically significant difference in CT group (*p* = 0.21).

#### 3.4.3 FMA

One study (38) reported on FMA, including 40 patients. The study evaluated two interventions: CT and AE. The AE group exhibited a significantly greater improvement in FMA scores (MD = 71.3, SD = 4.23) compared to the CT group (MD = 4.8, SD = 3.9). Statistical analysis indicated a significant difference in the AE group (p < 0.001), no statistically significant difference in CT group (*p* = 0.76).

### 3.5 Subacute phase

#### 3.5.1 10MWT

**Figure 4:**
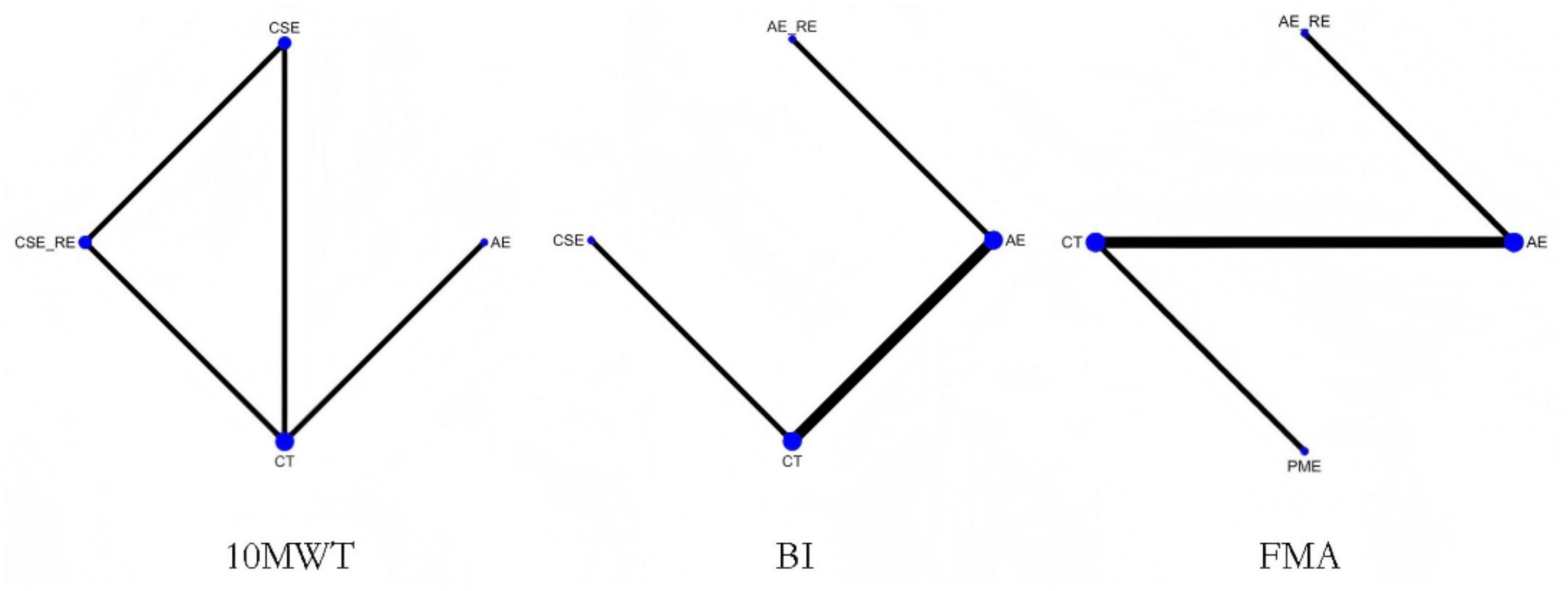
Network plot of the effectiveness of each intervention on 10MWT, BI, and FMA.

Three studies (41, 45, 47) reported on 10MWT, including 106 patients. Four interventions were assessed: CT, AE, CSE, and CSE + RE. Figure 4 displays the network plot for the 10MWT. An inconsistency model was leveraged to assess the overall inconsistency. The difference in DIC was 0.051, suggesting a minor difference. Therefore, the inconsistency was not notable, and a consistency model was selected. I^2^ statistic of 48%, indicating moderate heterogeneity in the network.

**Figure 5:**
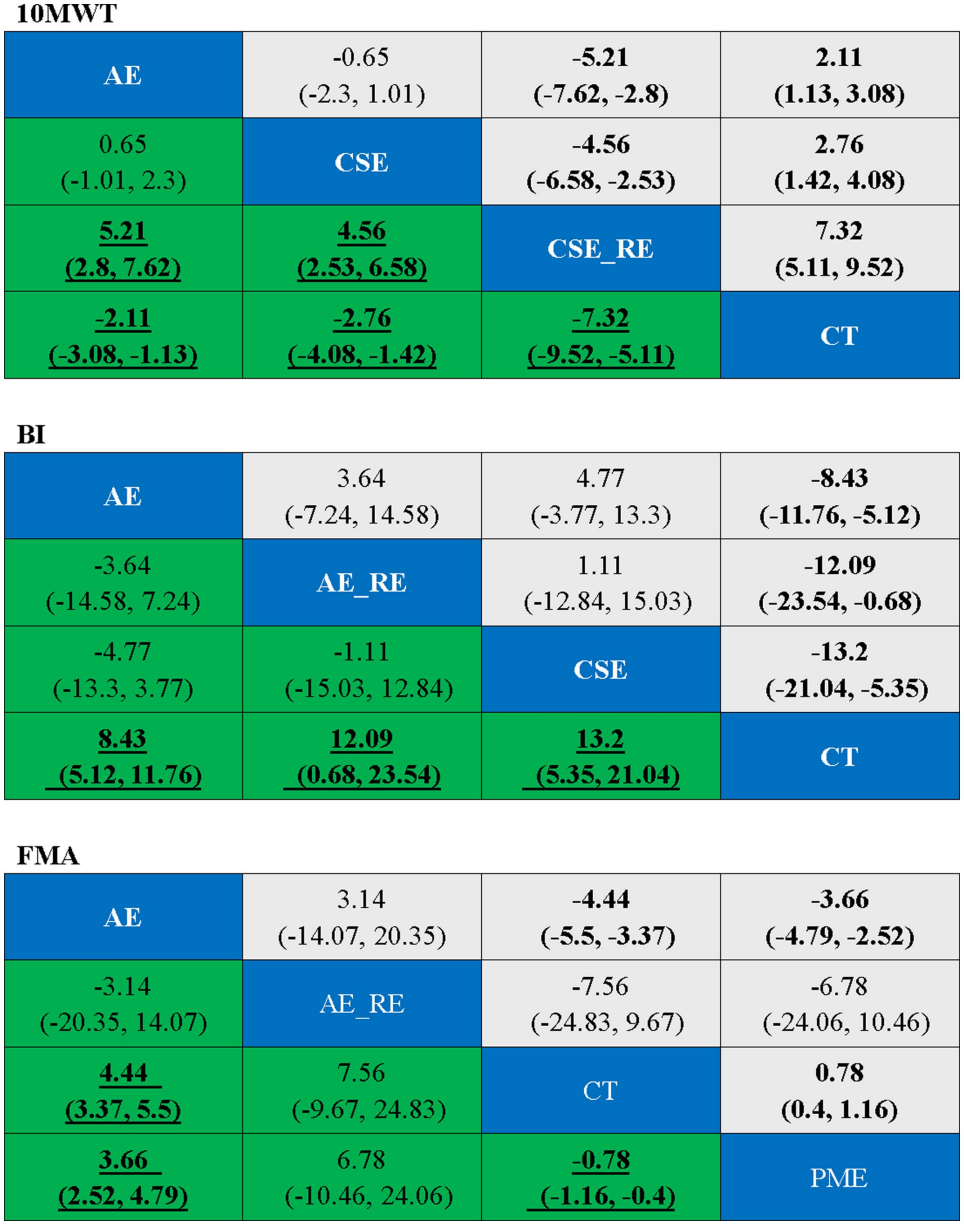
Ranking table for each outcome indicator, with bold underlining indicating significance.

**Figure 6:**
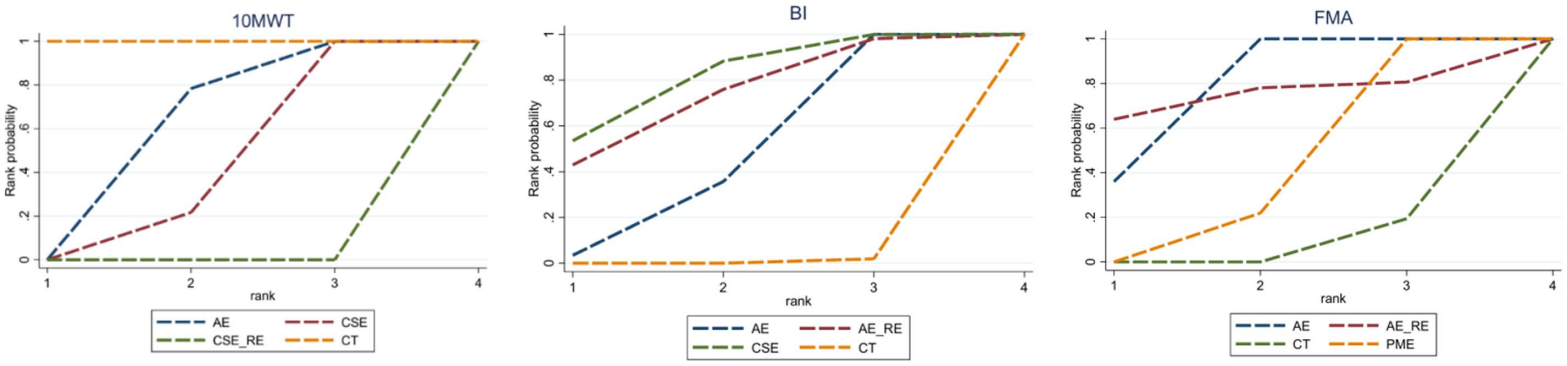
SUCRA values for each outcome indicator.

In the network meta-analysis (NMA), six comparisons were generated. CSE_RE (MD = −7.32, 95% CI [−9.52, −5.11]), CSE (MD = −2.76, 95% CI [−4.08, −1.42]), and AE (MD = −2.11, 95% CI [−3.08, −1.13]) demonstrated significantly greater improvements in 10MWT compared to CT. Moreover, CSE_RE (MD = −5.21, 95% CI [−7.62, −2.8]) exhibited a significantly greater effect than AE, and CSE_RE (MD = −4.56, 95% CI [−6.58, −2.53]) outperformed CSE. No significant differences were observed among the remaining groups (Figure 5). Based on the Surface Under the Cumulative Ranking (SUCRA), the ranking of interventions for improving 10MWT was as follows: CSE_RE (99.99%) > CSE (59.35%) > AE (40.65%) > CT (0.002%) (Figure 6; Table 3).

**Table 3:** SUCRA ranking of different intervention.

Interventions	10MWT		BI		FMA	
	SUCRA, %	Rank	SUCRA, %	Rank	SUCRA, %	Rank
CT	0.002	4	0.64	4	6.51	4
AE	40.65	3	46.42	3	78.73	1
AE + RE			72.10	2	74.07	2
CSE	59.35	2	80.84	1		
CSE+RE	99.99	1				
PME					40.69	3

A higher SUCRA value indicates a higher rank, which signifies a more favorable effect.

#### 3.5.2 BI

Four studies (42–44, 46) reported on BI, including 409 patients. Four interventions were assessed: CT, AE, CSE, and AE + RE. Figure 4 displays the network plot for the BI. An inconsistency model was leveraged to assess the overall inconsistency. The difference in DIC was 0.014, suggesting a minor difference. The inconsistency was not notable, and a consistency model was selected. I^2^ statistic of 62%, indicating high heterogeneity in the network. As the four interventions did not form a closed loop, the node-splitting method was not employed to assess local inconsistency.

In the NMA, six comparisons were generated. CSE (MD = 13.2, 95% CI [5.35, 21.04]), AE_RE (MD = 12.09, 95% CI [0.68, 23.54]), and AE (MD = 8.43, 95% CI [5.12, 11.76]) demonstrated significantly greater improvements in BI compared to CT. No significant differences were observed among the remaining groups (Figure 5). Based on SUCRA, the ranking of interventions for improving BI was as follows: CSE (80.84%) > AE_RE (72.10%) > AE (46.42%) > CT (0.64%) (Figure 6; Table 3).

#### 3.5.3 FMA

Four studies (40, 42, 44, 48) reported on FMA, including 342 patients. Four interventions were assessed: CT, AE, AE + RE, and PME. Figure 4 displays the network plot for the FMA. The inconsistency model analysis indicated a DIC difference of 0.011, suggesting a minor discrepancy. Therefore, the consistency model was selected. I^2^ statistic of 67%, indicating high heterogeneity in the network. As the four interventions did not form a closed loop, the node-splitting method was not applied to assess local inconsistency.

In the NMA, six comparisons were generated. AE (MD = 4.44, 95% CI [3.37, 5.5]) and PME (MD = 0.78, 95% CI [0.4, 1.16]) demonstrated significantly greater improvements in FMA compared to CT. Additionally, AE (MD = 3.66, 95% CI [2.52, 4.79]) showed significantly greater improvement than PME. No significant differences were observed among the remaining groups (Figure 5). Based on SUCRA, the ranking of interventions for improving FMA was as follows: AE (78.73%) > AE_RE (74.07%) > PME (40.69%) > CT (6.51%) (Figure 6; Table 3).

#### 3.5.4 6MWD

Two studies (43, 45) reported on 6MWD, including 228 patients and involving two interventions: CT and AE. In stud 1 (43), AE (MD = 68, SD = 118.81) and CT (MD = 46, SD = 114.01) were reported, though *p*-values were not provided. In study 2 (45), AE (MD = 105.1, SD = 79.5) and CT (MD = 35.9, SD = 115.1) were reported, with a significant between-group interaction *p*-value of 0.011. AE consistently demonstrated superior effectiveness in improving 6MWD compared to CT.

### 3.6 Chronic phase

#### 3.6.1 6MWD

**Figure 7:**
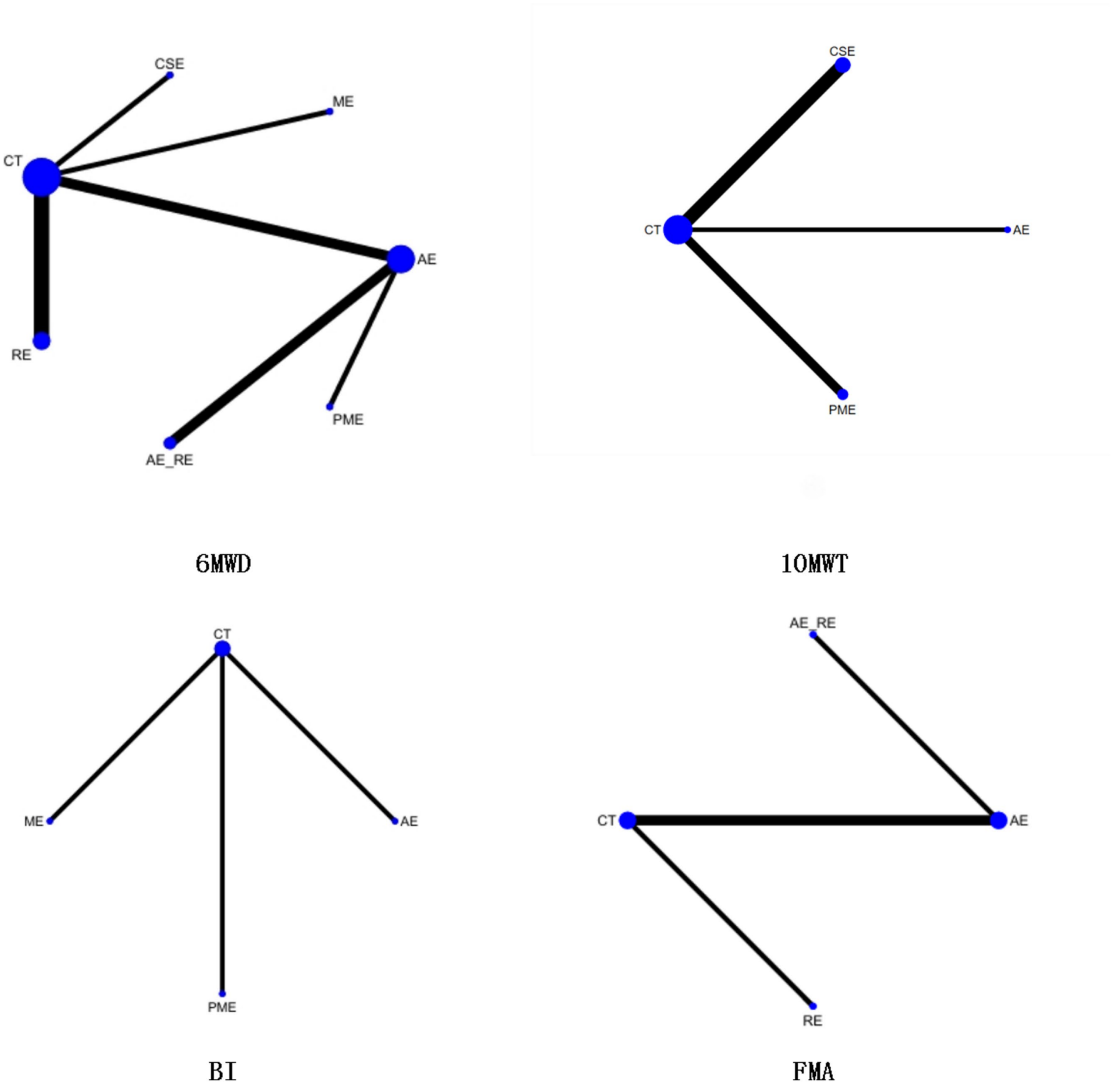
Network plot of the effectiveness of each intervention on 6MWD, 10MWT, BI, and FMA.

Twelve studies (49, 52–54, 58–60, 62, 64, 68–70) reported on 6MWD, including 441 patients. Seven interventions were assessed: CT, AE, AE + RE, CSE, PME, RE, and ME. Figure 7 displays the network plot of 6MWD. The inconsistency model analysis indicated a DIC difference of 0.036, suggesting a minor discrepancy. Therefore, the consistency model was adopted. As the seven interventions did not form a closed loop, the node-splitting method was not applied to assess local inconsistency.

**Figure 8:**
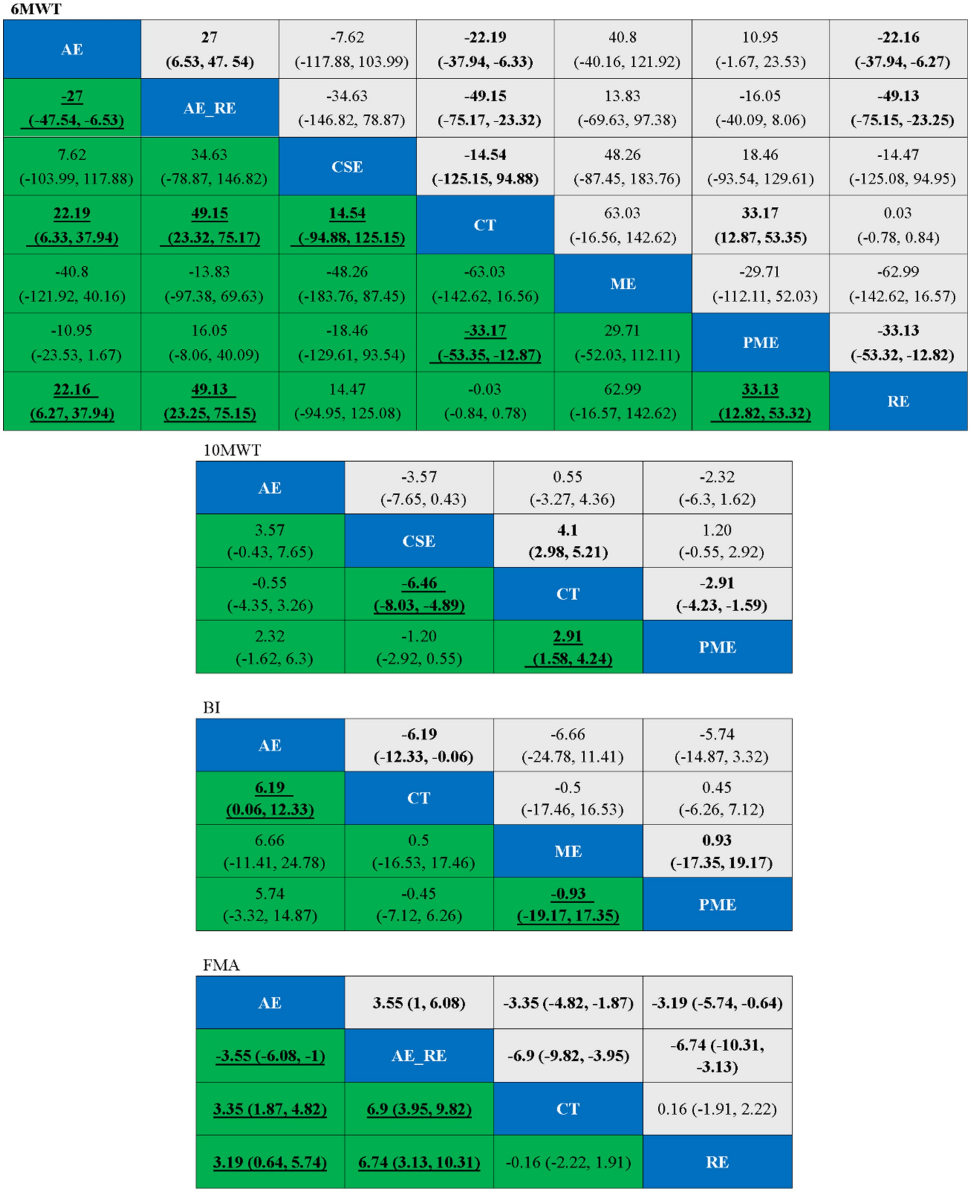
Ranking table for each outcome indicator, with bold underlining indicating significant differences.

**Figure 9:**
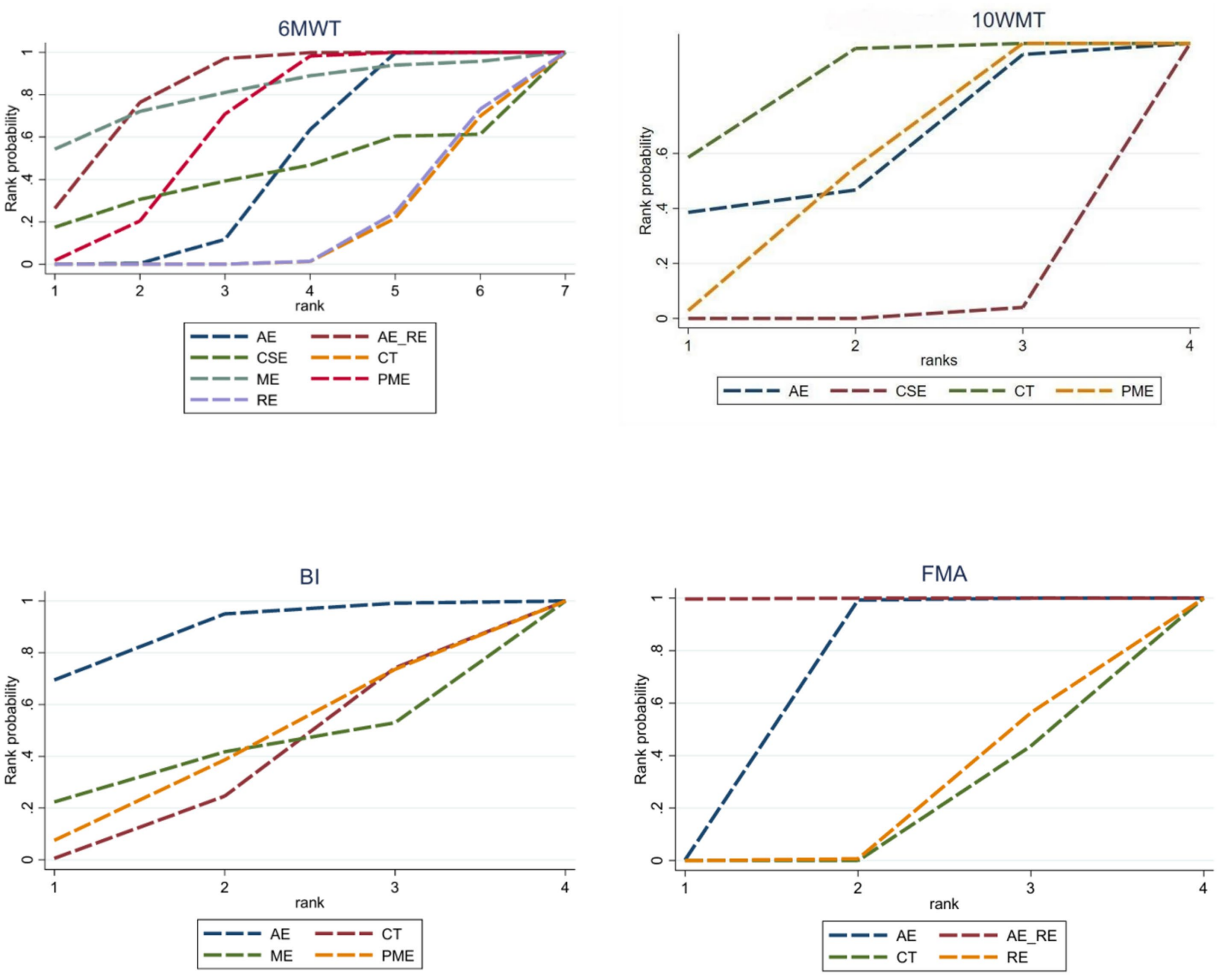
SUCRA values for each outcome indicator.

In the NMA, 21 comparisons were generated. AE + RE (MD = 49.15, 95% CI [23.32, 75.17]), PME (MD = 33.17, 95% CI [12.87, 53.35]), and AE (MD = 22.19, 95% CI [6.33, 37.94]) significantly improved 6MWD compared to CT. PME (MD = 33.13, 95% CI [12.82, 53.32]) and AE (MD = 22.16, 95% CI [6.27, 37.94]) also showed significantly superior effects compared to RE. AE + RE (MD = 49.13, 95% CI [23.25, 75.15]) demonstrated a significantly greater improvement than RE, while AE + RE (MD = 27, 95% CI [6.53, 47.54]) was significantly superior to AE. No significant differences were observed among the remaining groups (Figure 8). According to the Surface Under the Cumulative Ranking Curve (SUCRA), the ranking of interventions in terms of 6MWD improvement was as follows: AE + RE (83.33%) > ME (81.01%) > PME (65.26%) > AE (45.98%) > CSE (42.37%) > RE (16.54%) > CT (15.52%) (Figure 9; Table 4).

**Table 4:** SUCRA ranking of different intervention.

Interventions	6MWD		10MWT		BI		FMA	
	SUCRA, %	Rank	SUCRA, %	Rank	SUCRA, %	Rank	SUCRA, %	Rank
CT	15.52	7	14.42	4	33.16	4	14.69	4
AE	45.98	4	39.53	3	87.85	1	66.54	2
AE + RE	83.33	1					99.90	1
CSE	42.37	5	98.62	1				
PME	65.26	3	47.41	2	39.96	2		
RE	16.54	6					18.88	3
ME	81.01	2			39.03	3		

A higher SUCRA value indicates a higher rank, which signifies a more favorable effect.

#### 3.6.2 10MWT

Five studies (55, 56, 60, 61, 63, 72) reported on 10MWT, including 96 patients. Four interventions were assessed: CT, AE, PME, and CSE. Figure 7 displays the network plot for the 10MWT. The inconsistency model analysis indicated a DIC difference of 0.076, suggesting a minor discrepancy. Therefore, the consistency model was selected. I^2^ statistic of 43%, indicating moderate heterogeneity in the network. As the four interventions did not form a closed loop, the node-splitting method was not applied to assess local inconsistency.

In the NMA, six comparisons were generated. CSE (MD = −6.46, 95% CI [−8.03, −4.89]) and PME (MD = 2.91, 95% CI [1.58, 4.24]) significantly improved 10MWT compared to CT, while no significant differences were found among the remaining groups (Figure 8). According to SUCRA, the ranking of interventions for improving 10MWT was: CSE (98.62%) > PME (47.41%) > AE (39.53%) > CT (14.42%) (Figure 9; Table 4).

#### 3.6.3 BI

Four studies (50, 51, 65, 66) reported on BI, including 131 patients. Four interventions assessed: CT, AE, PME, and ME. Figure 7 displays the network plot for the BI. The inconsistency model analysis indicated a DIC difference of 0.022, suggesting a minor discrepancy. Therefore, the consistency model was selected. I^2^ statistic of 72%, indicating high heterogeneity in the network. As the four interventions did not form a closed loop, the node-splitting method was not applied to assess local inconsistency.

In the NMA, six comparisons were generated. AE (MD = 6.19, 95% CI [0.06, 12.33]) BI significantly improved compared to CT, while no significant differences were found among the remaining groups (Figure 8). According to SUCRA, the ranking of interventions for improving BI was: AE (87.85%) > PME (39.96%) > ME (39.03%) > CT (33.16%) (Figure 9; Table 4).

#### 3.6.4 FMA

Four studies (51, 57, 67, 71) reported on FMA, including 105 patients. Four interventions assessed: CT, AE, RE, and AE + RE. Figure 7 displays the network plot for the FMA. The inconsistency model analysis indicated a DIC difference of 0.034, suggesting a minor discrepancy. Therefore, the consistency model was selected. I^2^ statistic of 58%, indicating high heterogeneity in the network. As the four interventions did not form a closed loop, the node-splitting method was not applied to assess local inconsistency.

In the NMA, six comparisons were generated. AE + RE (MD = 6.9, 95% CI [3.95, 9.82]) and AE (MD = 3.35, 95% CI [1.87, 4.82]) significantly improved FMA compared to CT. Additionally, AE + RE (MD = 6.74, 95% CI [3.13, 10.31]) and AE (MD = 3.19, 95% CI [0.64, 5.74]) exhibited significantly superior effects compared to RE. AE + RE (MD = 3.55, 95% CI [1, 6.08]) was also significantly more effective than AE, while no significant differences were observed among the remaining groups (Figure 8). According to SUCRA, the ranking of interventions for improving FMA was: AE + RE (99.90%) > AE (66.54%) > RE (18.88%) > CT (14.69%) (Figure 9; Table 4).

### 3.7 Publication bias analysis

**Figure 10:**
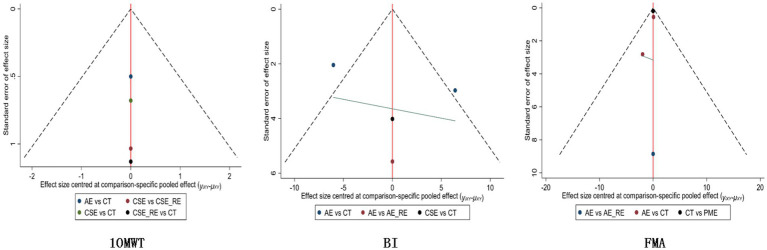
Publication bias for each outcome indicator in the subacute phase.

**Figure 11:**
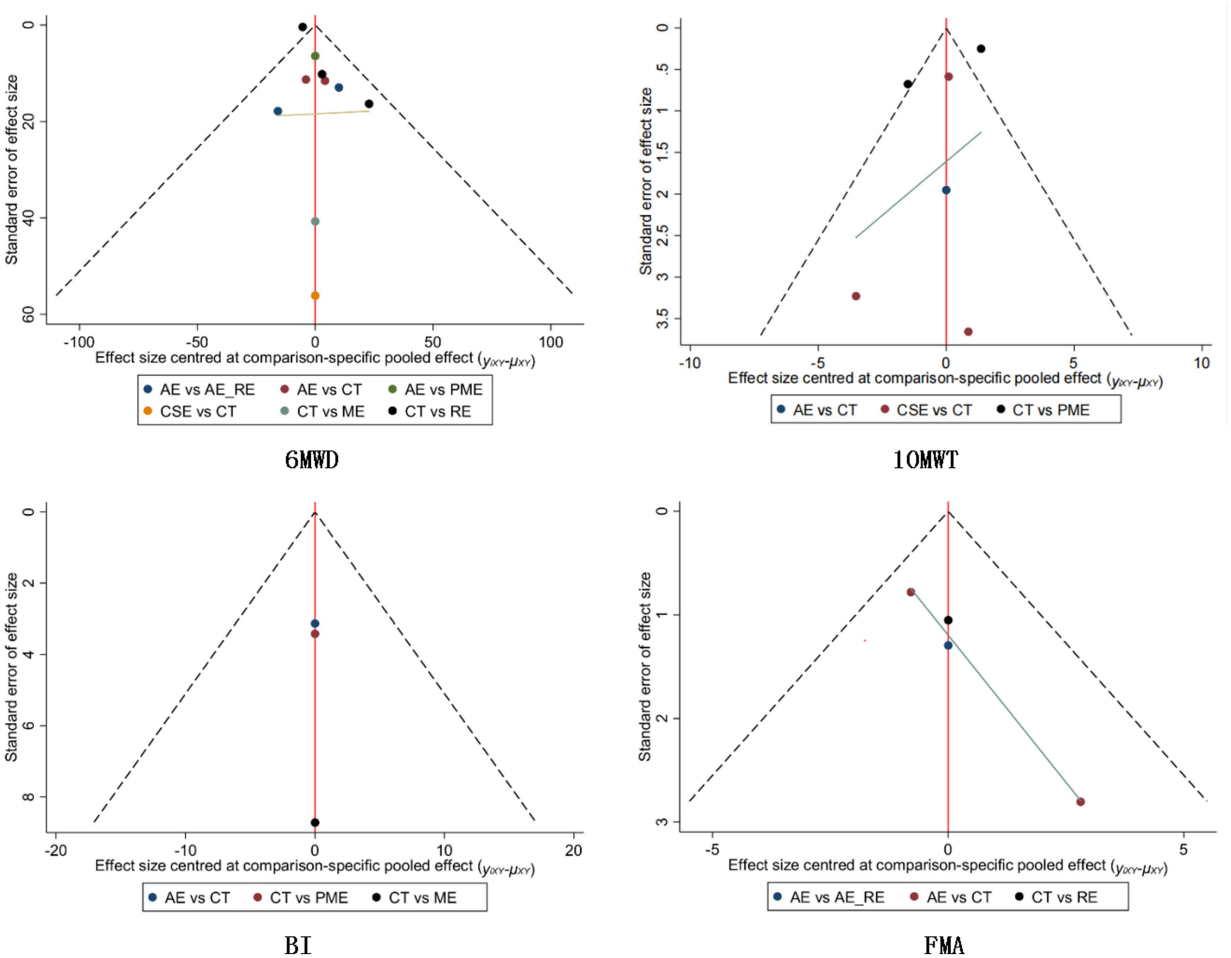
Publication bias for each outcome indicator in the chronic phase.

In the funnel plot showing publication bias, each colored dot represents a pairwise comparison between different interventions. The number of dots increases as the frequency of pairwise comparisons increases. The symmetrical distribution of points in the funnel plot indicates a lower risk of publication bias. In Figures 10, 11, almost all of the points are in the inner measurements of the funnel plot, and most of them are symmetrically distributed, which suggests a lower publication bias.

### 3.8 Results of meta-regression

We conducted a meta-regression to examine the moderating effects of age and intervention duration on the efficacy of different exercise interventions. The results are summarized in Supplementary Table S2. The analysis revealed that intervention duration was a significant moderator. For improvement in the 6-min walk distance (6MWD), longer intervention duration was associated with greater treatment benefits for both aerobic exercise (AE) (β = 3.927, 95% CrI: 0.891 to 6.963) and mixed exercise (ME) (β = 4.215, 95% CrI: 1.028 to 7.402) compared to conventional therapy (CT). Similarly, for improvement in the 10-meter walk test (10MWT), intervention duration showed a significant positive moderating effect for AE vs. CT (β = 1.927, 95% CrI: 0.491 to 3.363) and combined strength and endurance exercise (CSE) vs. CT (β = 2.415, 95% CrI: 0.728 to 4.102). Regarding improvements in the Barthel Index (BI) and Fugl-Meyer Assessment (FMA), intervention duration also demonstrated significant positive associations across multiple comparisons, including AE vs. CT and CSE vs. CT. In contrast, patient age did not exhibit a statistically significant moderating effect for any outcome measure or in the vast majority of intervention comparisons (95% CrI included zero). The residual heterogeneity across regression models was low (I^2^ between 8 and 16%), suggesting that the covariates of age and intervention duration accounted for some of the observed heterogeneity, while the overall models remained robust.

### 3.9 Assessment of the confidence in the evidence

The confidence in the evidence from the network meta-analysis was assessed using the CINeMA framework. Overall, for all four primary outcomes, the evidence for most pairwise comparisons was rated as low or very low. Only a few comparisons were assessed as moderate, and just two were high quality. (1) 6-Minute Walk Test: In analyses involving direct comparison evidence, the confidence was primarily low or very low. The main reasons for downgrading were considerable heterogeneity and inconsistency. For many intervention contrasts lacking direct comparisons (number of studies = 0), the confidence was also low or very low, primarily limited by serious imprecision. (2) 10-Meter Walk Test: The confidence in the evidence ranged from high to very low. The comparison “CSE_RE: CT” received a high confidence rating. However, for most comparisons, the confidence was moderate or low, with downgrading factors mainly including imprecision and some risk of bias. (3) Barthel Index: The confidence for all comparisons was low or very low. The most dominant factor leading to downgrading was serious imprecision, followed by inconsistency and risk of bias. (4) Fugl-Meyer Assessment: The confidence was primarily low and very low. The main drivers for downgrading were serious imprecision and some risk of bias.

Detailed CINeMA assessment results, including the specific judgments across the six domains for each comparison, are provided in Supplementary Table S3.

## 4 Discussion

Our CINeMA evaluation indicates that the evidence supporting most comparisons in this network meta-analysis is of low or very low quality. This is primarily attributable to the limited number of included studies (leading to imprecision) and the presence of heterogeneity across studies. Therefore, inferences regarding the relative superiority of interventions based on SUCRA rankings should be considered preliminary and suggestive—serving to guide future research rather than to inform definitive clinical decision-making. The present systematic review synthesizes the available evidence on the effects of different exercise interventions on motor function in patients at different phases of recovery post-stroke. Findings suggest that in the acute phase, aerobic exercise (AE) may offer superior benefits over conventional therapy (CT) in enhancing endurance, improving limb motor control, and promoting activities of daily living (ADL). During the subacute phase, core stability exercise combined with resistance exercise (CSE + RE) appears to be the most effective approach for improving walking ability, whereas standalone core stability exercise (CSE) is more advantageous for enhancing ADL performance. AE also remains favorable in augmenting endurance and refining motor control in this phase. In the chronic phase, CSE shows greater efficacy in improving walking ability, AE demonstrates superior effects in facilitating ADL, and combined AE and resistance exercise (AE + RE) yields the most significant improvements in both endurance and motor control.

### 4.1 Effects of exercise interventions on walking ability

The subacute phase is the critical window during which neuroplasticity is most active in post-stroke patients, when neural adaptations in the brain and spinal cord are enhanced and are particularly sensitive in response to exercise training (73). The superior intervention effect of CSE + RE at this phase may be attributed to the ability of resistance exercise (RE) to effectively enhance lower limb strength, increase walking speed, and improve gait stability (74). Secondly, the training modality is effective in improving stride length and gait symmetry, thereby enhancing walking quality (75). In addition, CSE prompts patients to adjust their gait patterns in real time in complex environments, further reducing the risk of falls (76).

As the patients enter the chronic phase, their neuroplasticity decreases and the recovery of motor function stabilizes. The enhancement of walking ability is predominantly contingent on stability and functional compensatory mechanisms during ambulation, rather than on plyometric training in isolation (77). It is challenging for patients to achieve substantial enhancements in ambulatory function through RE at this phase, owing to diminished neuroadaptation. Consequently, CSE emerges as a more appealing intervention (78, 79).

### 4.2 Effects of exercise interventions on activities of daily living

The acute phase marks the initial stage of neurological recovery in post-stroke patients, during which they typically experience severe neurological impairments, reduced cardiopulmonary endurance, and physical deconditioning. At this phase, restoring the ability to perform ADL hinges on improving systemic endurance, enhancing cardiovascular function, and facilitating neuroplasticity (80). The considerable potential of AE at this phase may be attributed to its significant enhancement of cardiopulmonary endurance, which in turn improves patients’ physical activity capacity and accelerates the recovery of independence in activities of daily living (81). Additionally, AE has been shown to increase cerebral blood flow, promote neuroplastic changes, and expedite motor function recovery (43). Beyond its physiological benefits, AE also helps alleviate fatigue, improve mood, and enhance overall quality of life, which collectively foster greater engagement in rehabilitation activities (82).

As the patient enters the subacute phase, neuroplasticity increases, and motor function is gradually restored. Inadequate core control and unstable balance become significant factors in the limitation of ADL. AE has limited improvement in gait and postural control at this time (83). CSE is emerging as a better intervention. It has been demonstrated to enable patients to perform daily activities more safely and efficiently by enhancing the stability of trunk muscle groups, optimizing postural control, improving balance, and reducing the risk of falls (78).

In the chronic phase, the primary role of AE shifts from promoting neuroplasticity and restoring cardiorespiratory endurance to counteracting long-term physical deconditioning, optimizing exercise endurance, and modifying compensatory movement patterns (84). Unlike acute-phase interventions, chronic-phase AE prioritizes gait optimization and compensatory strategy adjustments. These adaptations reduce reliance on the unaffected limb, improve walking symmetry, and enhance independent ambulation (85). Furthermore, a core objective of chronic-phase AE is to prevent declines in exercise tolerance and daily activity capacity, enabling patients to sustain higher functional independence for familial and societal participation (86). By emphasizing long-term functional maintenance and gait quality over isolated cardiorespiratory adaptations, chronic-phase AE promotes sustained and efficient engagement in daily living.

### 4.3 Effects of exercise interventions on exercise endurance and limb control

During the acute phase of stroke, motor endurance declines significantly, accompanied by impaired limb control, primarily due to the combined effects of neurological injury and metabolic disturbances. Studies have shown that patients in this phase experience a sharp deterioration in cardiorespiratory fitness, characterized by a marked reduction in maximal oxygen uptake (VO₂ max). Clinically, this manifests as muscle weakness, abnormal muscle tone, and impaired coordination, all of which further restrict voluntary motor activity (87). Additionally, prolonged bed rest and sedentary behavior commonly lead to disuse muscle atrophy, which exacerbates the decline in endurance and further impairs motor control (88). In this phase, AE has demonstrated distinct rehabilitative advantages as an early intervention. AE effectively enhances motor endurance by improving VO₂ max and cardiopulmonary adaptability (89). Moreover, it increases skeletal muscle capillary density and optimizes oxygen delivery, thereby further enhancing muscular endurance (90). Importantly, AE also promotes neuroplasticity by upregulating brain-derived neurotrophic factor (BDNF), which facilitates synaptic plasticity and motor cortex remodeling. These mechanisms provide a physiological foundation for restoring limb control (8). Therefore, early implementation of AE in the acute phase not only improves motor endurance and limb function but also enhances neural adaptability, laying a solid groundwork for subsequent rehabilitation.

During the subacute phase, patients gradually regain voluntary motor function; however, motor endurance and limb control remain impaired due to incomplete neuromuscular adaptation. At this phase, AE continues to serve as the primary intervention, with its role in optimizing mitochondrial function being particularly important. Studies have demonstrated that AE enhances exercise endurance by activating PGC-1α–mediated mitochondrial biogenesis, improving oxidative phosphorylation, and increasing ATP production efficiency (91). Furthermore, AE promotes capillary formation, enhances oxygen delivery to skeletal muscles, and improves microcirculatory function, thereby contributing to further improvements in muscular endurance (92).

Upon entering the chronic phase, patients’ exercise endurance generally stabilizes; however, muscle atrophy, shifts in muscle fiber composition, and reduced neural adaptability become the principal barriers to further functional recovery (93). Relying solely on AE is insufficient to address these issues, making the AE + RE a more effective intervention. RE promotes myofibrillar protein synthesis via the mTOR-dependent signaling pathway, increases the proportion of type IIa muscle fibers (fatigue-resistant), and decreases type IIx fibers (low-endurance, fast-twitch), thereby enhancing both muscular strength and endurance (94). Moreover, RE optimizes motor unit recruitment patterns, improves neural conduction efficiency, and enhances neuromuscular coordination, contributing to better fine motor control post-stroke (89). When combined with AE, it further improves energy metabolism and muscular endurance (95). However, some studies suggest that prematurely introducing high-intensity RE in chronic stroke patients may elevate neural load, potentially compromising neuroplasticity and the stability of motor recovery (96). Therefore, individualized adjustments to training intensity and a well-balanced ratio of AE to RE are essential to meet the dual goals of enhancing endurance and supporting neural adaptation.

### 4.4 Clinical significance and practical implications

The findings of this study provide phased and individualized evidence-based guidance for the clinical practice of motor rehabilitation post-stroke. Based on the NMA results, we propose the following practical recommendations for clinicians and therapists to optimize exercise prescription: (1) Acute Phase (0–7 days): The primary goals are to improve overall endurance, promote neuroplasticity, and facilitate the early restoration of Activities of Daily Living (ADL). The preferred exercise intervention is Aerobic Exercise (AE), such as bedside cycling or low-intensity upper limb ergometer training. Exercise should commence at low-to-moderate intensity (e.g., Borg Scale 11–13) for short durations (e.g., 10–15 min per session), with close monitoring of vital signs. The emphasis is on “initiating movement” to break the vicious cycle of bed rest and lay the cardiopulmonary and neurological foundation for subsequent rehabilitation. (2) Subacute Phase (7 days – 6 months): The main objectives are to specifically enhance walking capacity, balance function, and independence in ADL. Core Stability Exercises (CSE) combined with Resistance Exercise (RE) are recommended as the priority. For instance, leg strength training (e.g., elastic band resisted hip flexion and knee extension) can be combined with exercises like planks and bridge exercises to activate core muscles, thereby synergistically improving gait stability and efficiency. CSE demonstrated the greatest advantage in improving ADL. This phase should focus on training the patient’s trunk control in dynamic sitting and standing positions, which is crucial for performing daily tasks such as dressing and transferring safely and efficiently. (3) Chronic Phase (≥6 months): This phase aims to further enhance endurance and quality of motor control, optimize compensatory strategies, and promote community reintegration. For improving endurance and motor control, a combination of Aerobic Exercise and Resistance Exercise (AE + RE) is likely the optimal approach. For example, a weekly plan could include 2–3 sessions of brisk walking or stationary cycling (AE), combined with 1–2 sessions of strength training for both the affected and unaffected limbs (RE), to achieve dual improvements in cardiopulmonary function and muscle strength, which are vital for long-term functional independence. For improving walking capacity, Core Stability Exercises (CSE) were most effective. Training at this stage should be more functional, such as walking on uneven surfaces or while carrying objects, to simulate real-world environments. For improving ADL, Aerobic Exercise (AE) remains the preferred choice, with the goal shifting toward maintaining the overall stamina and energy required for participation in household and community activities.

In clinical practice, therapists should utilize the evidence from this study, integrated with the patient’s specific functional assessment results (e.g., FMA, BI scores), personal goals, comorbidities, and preferences, to formulate an “individualized prescription.” For instance, for a subacute patient whose primary goal is to resume community walking, the prescription might prioritize CSE + RE, supplemented with a minor AE component to maintain endurance.

### 4.5 Limitations and future directions

This study divides the recovery process into the acute phase (0–7 days), subacute phase (7 days to 6 months), and chronic phase (≥6 months) based on changes in post-stroke neuroplasticity. However, this temporal classification is not absolute, as patients’ recovery trajectories may vary due to individual differences such as age, comorbidities, stroke type, and severity. With ongoing advances in medical science and mechanistic research, a more refined and precise phase classification system is anticipated in the future.

It is noteworthy that this study included a limited number of studies addressing the acute and subacute phases, with interventions primarily focusing on AE and CT. Some of the results did not reach statistical significance. The small sample sizes and variability in intervention protocols limit the generalizability of the findings. Furthermore, the absence of long-term follow-up hinders the evaluation of sustained recovery and the prevention of stroke recurrence during the chronic phase. Additionally, individual factors such as physical characteristics, lesion location, and psychological status were not sufficiently accounted for.

Furthermore, our study has two additional notable limitations. First, none of the included RCTs reported outcome data beyond the immediate post-intervention period (typically less than 6 months). Consequently, our findings are unable to elucidate the long-term durability and sustainability of the observed exercise benefits across different recovery phases. Second, adverse events associated with exercise interventions were not systematically reported or consistently defined across the majority of trials. This gap in reporting prevents a comprehensive assessment of the safety profiles and potential risks of these interventions, which is a critical consideration for their implementation in clinical practice.

Despite these limitations, this study provides a theoretical basis for a phased exercise intervention. Future research should prioritize long-term follow-up assessments to determine the persistence of treatment effects and the potential for preventing stroke recurrence. Additionally, standardized monitoring and reporting of adverse events in future trials are essential to establish the safety and risk–benefit ratio of these exercise regimens, thereby facilitating the development of safer and more effective, personalized rehabilitation protocols.

### 4.6 Conclusion

The comprehensive evidence provided by this study indicates that different types of exercise interventions have significantly different effects on motor function across various post-stroke recovery phases. Aerobic exercise (AE) appears to be most suitable during the acute phase, while core stability exercise combined with resistance exercise (CSE + RE) demonstrates optimal effectiveness in the subacute phase. In the chronic phase, the combination of aerobic and resistance exercise (AE + RE) shows a significant advantage. Personalized and phase-specific interventions are crucial for motor function recovery, although the long-term effects and optimization of individualized programs require further investigation.

## Data Availability

The original contributions presented in the study are included in the article/Supplementary material, further inquiries can be directed to the corresponding authors.
