# Supplementary material for: Effects of different exercise interventions on motor function in patients at different post-stroke recovery phases: a systematic review and Bayesian network meta-analysis
Source: Front Neurol. 2025 Dec 8;16:1678951. doi: 10.3389/fneur.2025.1678951 (PMC12719286; doi:10.3389/fneur.2025.1678951)

**Table S1:** Search Strategies

S1.1 Search Strategies for Web of Science

| NO. | Query |
| --- | --- |
| #1 | stroke (Topic) |
| #2 | TS=('Strokes' OR 'Cerebrovascular Accident*' OR 'Cerebral Stroke*' OR 'Cerebrovascular Apoplexy' OR 'Brain Vascular Accident*' OR 'Cerebrovascular Stroke*' OR 'Apoplexy' OR 'CVA' OR 'CVAs' OR 'Cerebrovascular Accident' OR 'Acute Stroke*' OR 'Acute Cerebrovascular Accident*' OR 'acute cerebrovascular lesion' OR 'acute focal cerebral vasculopathy' OR 'apoplectic stroke' OR 'apoplexia' OR 'brain accident' OR 'brain attack' OR 'brain blood flow disturbance' OR 'brain insult' OR 'brain insultus' OR 'cerebral apoplexia' OR 'cerebral insult' OR 'cerebral vascular accident' OR 'cerebral vascular insufficiency' OR 'cerebro vascular accident' OR 'cerebrovascular arrest' OR 'cerebrovascular failure' OR 'cerebrovascular injury' OR 'cerebrovascular insufficiency' OR 'cerebrovascular insult' OR 'cerebrum vascular accident' OR 'cryptogenic stroke' OR 'insultus cerebralis' OR 'ischaemic seizure' OR 'ischemic seizure' OR 'stroke' OR 'thrombotic stroke') |
| #3 | #1 OR #2 |
| #4 | TS=(exercise) |
| #5 | TS=('Exercises' OR 'Physical Exercise*' OR 'Physical Activity*' OR 'Aerobic Exercise*' OR 'Isometric Exercise*' OR 'Acute Exercise*' OR 'Exercise Training*' OR 'biometric exercise' OR 'effort' OR 'exercise capacity' OR 'exercise performance' OR 'exertion' OR 'fitness training' OR 'fitness workout' OR 'physical effort' OR 'physical exertion' OR 'physical workout' OR 'exercise') |
| #6 | #4 OR #5 |
| #7 | TS=(Resistance Training) |
| #8 | TS=('Strength Training' OR 'Weight Lifting Strengthening Program*' OR 'Weight Lifting Exercise Program*' OR 'Weight Bearing Strengthening Program*' OR 'Weight Bearing Exercise Program*' OR 'resistance exercise' OR 'resistance exercise training' OR 'resistance-type exercise' OR 'resistance-type training' OR 'strength-type exercise' OR 'strength-type training' OR 'resistance training') |
| #9 | #7 OR #8 |
| #10 | TS=(High-Intensity Interval Training) |
| #11 | TS=('High Intensity Interval Training*' OR 'High-Intensity Intermittent Exercise*' OR 'Sprint Interval Training*' OR 'high-intensity intermittent training' OR 'high-intensity interval exercise' OR 'high-intensity interval training' OR 'HIIE' OR 'HIIT' OR 'intermittent high-intensity training' OR 'interval high-intensity training') |
| #12 | #10 OR #11 |
| #13 | #6 OR #9 OR #12 |
| #14 | #3 AND #13 |
| #15 | TS=(Randomized Controlled Trial) |
| #16 | TS=('controlled trial, randomized' OR 'randomised controlled study' OR 'randomised controlled trial' OR 'randomized controlled study' OR 'randomized controlled trial' OR 'rct' OR 'RCT') |
| #17 | #15 OR #16 |
| #18 | #14 AND #17 |

S1.2 Search Strategies for Embase

| No. | Query |
| --- | --- |
| #16 | #14 AND #15 |
| #15 | 'randomized controlled trial'/exp |
| #14 | #3 AND #13 |
| #13 | #6 OR #9 OR #12 |
| #12 | #10 OR #11 |
| #11 | 'high intensity interval training*':ab,ti OR 'high-intensity intermittent exercise*':ab,ti OR 'sprint interval training*':ab,ti OR 'high-intensity intermittent training':ab,ti OR 'high-intensity interval exercise':ab,ti OR 'high-intensity interval training':ab,ti OR 'hiie':ab,ti OR 'hiit':ab,ti OR 'intermittent high-intensity training':ab,ti OR 'interval high-intensity training':ab,ti |
| #10 | 'high intensity interval training'/exp |
| #9 | #7 OR #8 |
| #8 | 'strength training':ab,ti OR 'weight lifting strengthening program*':ab,ti OR 'weight lifting exercise program*':ab,ti OR 'weight bearing strengthening program*':ab,ti OR 'weight bearing exercise program*':ab,ti OR 'resistance exercise':ab,ti OR 'resistance exercise training':ab,ti OR 'resistance-type exercise':ab,ti OR 'resistance-type training':ab,ti OR 'strength-type exercise':ab,ti OR 'strength-type training':ab,ti OR 'resistance training':ab,ti |
| #7 | 'resistance training'/exp |
| #6 | #4 OR #5 |
| #5 | 'exercises':ab,ti OR 'physical exercise*':ab,ti OR 'physical activit*':ab,ti OR 'aerobic exercise*':ab,ti OR 'isometric exercise*':ab,ti OR 'acute exercise*':ab,ti OR 'exercise training*':ab,ti OR 'biometric exercise':ab,ti OR 'effort':ab,ti OR 'exercise capacity':ab,ti OR 'exercise performance':ab,ti OR 'exertion':ab,ti OR 'fitness training':ab,ti OR 'fitness workout':ab,ti OR 'physical effort':ab,ti OR 'physical exertion':ab,ti OR 'physical workout':ab,ti OR 'exercise':ab,ti |
| #4 | 'exercise'/exp |
| #3 | #1 OR #2 |
| #2 | 'strokes':ab,ti OR 'cerebrovascular accident*':ab,ti OR 'cerebral stroke*':ab,ti OR 'cerebrovascular apoplexy':ab,ti OR 'brain vascular accident*':ab,ti OR 'cerebrovascular stroke*':ab,ti OR 'apoplexy':ab,ti OR 'cva':ab,ti OR 'cvas':ab,ti OR 'cerebrovascular accident':ab,ti OR 'acute stroke*':ab,ti OR 'acute cerebrovascular accident*':ab,ti OR 'acute cerebrovascular lesion':ab,ti OR 'acute focal cerebral vasculopathy':ab,ti OR 'apoplectic stroke':ab,ti OR 'apoplexia':ab,ti OR 'brain accident':ab,ti OR 'brain attack':ab,ti OR 'brain blood flow disturbance':ab,ti OR 'brain insult':ab,ti OR 'brain insultus':ab,ti OR 'cerebral apoplexia':ab,ti OR 'cerebral insult':ab,ti OR 'cerebral vascular accident':ab,ti OR 'cerebral vascular insufficiency':ab,ti OR 'cerebro vascular accident':ab,ti OR 'cerebrovascular arrest':ab,ti OR 'cerebrovascular failure':ab,ti OR 'cerebrovascular injury':ab,ti OR 'cerebrovascular insufficiency':ab,ti OR 'cerebrovascular insult':ab,ti OR 'cerebrum vascular accident':ab,ti OR 'cryptogenic stroke':ab,ti OR 'insultus cerebralis':ab,ti OR 'ischaemic seizure':ab,ti OR 'ischemic seizure':ab,ti OR 'stroke':ab,ti OR 'thrombotic stroke':ab,ti |
| #1 | 'cerebrovascular accident'/exp |

S1.3 Search Strategies for PubMed

| NO. | Query |
| --- | --- |
| #1 | "Stroke"[Mesh] |
| #2 | 'Strokes'[Title/Abstract] OR 'Cerebrovascular Accident*'[Title/Abstract] OR 'Cerebral Stroke*'[Title/Abstract] OR 'Cerebrovascular Apoplexy'[Title/Abstract] OR 'Brain Vascular Accident*'[Title/Abstract] OR 'Cerebrovascular Stroke*'[Title/Abstract] OR 'Apoplexy'[Title/Abstract] OR 'CVA'[Title/Abstract] OR 'CVAs'[Title/Abstract] OR 'Cerebrovascular Accident'[Title/Abstract] OR 'Acute Stroke*'[Title/Abstract] OR 'Acute Cerebrovascular Accident*'[Title/Abstract] OR 'acute cerebrovascular lesion'[Title/Abstract] OR 'acute focal cerebral vasculopathy'[Title/Abstract] OR 'apoplectic stroke'[Title/Abstract] OR 'apoplexia'[Title/Abstract] OR 'brain accident'[Title/Abstract] OR 'brain attack'[Title/Abstract] OR 'brain blood flow disturbance'[Title/Abstract] OR 'brain insult'[Title/Abstract] OR 'brain insultus'[Title/Abstract] OR 'cerebral apoplexia'[Title/Abstract] OR 'cerebral insult'[Title/Abstract] OR 'cerebral vascular accident'[Title/Abstract] OR 'cerebral vascular insufficiency'[Title/Abstract] OR 'cerebro vascular accident'[Title/Abstract] OR 'cerebrovascular arrest'[Title/Abstract] OR 'cerebrovascular failure'[Title/Abstract] OR 'cerebrovascular injury'[Title/Abstract] OR 'cerebrovascular insufficiency'[Title/Abstract] OR 'cerebrovascular insult'[Title/Abstract] OR 'cerebrum vascular accident'[Title/Abstract] OR 'cryptogenic stroke'[Title/Abstract] OR 'insultus cerebralis'[Title/Abstract] OR 'ischaemic seizure'[Title/Abstract] OR 'ischemic seizure'[Title/Abstract] OR 'stroke'[Title/Abstract] OR 'thrombotic stroke'[Title/Abstract] |
| #3 | ("Stroke"[Mesh]) OR ('Strokes'[Title/Abstract] OR 'Cerebrovascular Accident*'[Title/Abstract] OR 'Cerebral Stroke*'[Title/Abstract] OR 'Cerebrovascular Apoplexy'[Title/Abstract] OR 'Brain Vascular Accident*'[Title/Abstract] OR 'Cerebrovascular Stroke*'[Title/Abstract] OR 'Apoplexy'[Title/Abstract] OR 'CVA'[Title/Abstract] OR 'CVAs'[Title/Abstract] OR 'Cerebrovascular Accident'[Title/Abstract] OR 'Acute Stroke*'[Title/Abstract] OR 'Acute Cerebrovascular Accident*'[Title/Abstract] OR 'acute cerebrovascular lesion'[Title/Abstract] OR 'acute focal cerebral vasculopathy'[Title/Abstract] OR 'apoplectic stroke'[Title/Abstract] OR 'apoplexia'[Title/Abstract] OR 'brain accident'[Title/Abstract] OR 'brain attack'[Title/Abstract] OR 'brain blood flow disturbance'[Title/Abstract] OR 'brain insult'[Title/Abstract] OR 'brain insultus'[Title/Abstract] OR 'cerebral apoplexia'[Title/Abstract] OR 'cerebral insult'[Title/Abstract] OR 'cerebral vascular accident'[Title/Abstract] OR 'cerebral vascular insufficiency'[Title/Abstract] OR 'cerebro vascular accident'[Title/Abstract] OR 'cerebrovascular arrest'[Title/Abstract] OR 'cerebrovascular failure'[Title/Abstract] OR 'cerebrovascular injury'[Title/Abstract] OR 'cerebrovascular insufficiency'[Title/Abstract] OR 'cerebrovascular insult'[Title/Abstract] OR 'cerebrum vascular accident'[Title/Abstract] OR 'cryptogenic stroke'[Title/Abstract] OR 'insultus cerebralis'[Title/Abstract] OR 'ischaemic seizure'[Title/Abstract] OR 'ischemic seizure'[Title/Abstract] OR 'stroke'[Title/Abstract] OR 'thrombotic stroke'[Title/Abstract]) |
| #4 | "Exercise"[Mesh] |
| #5 | 'Exercises'[Title/Abstract] OR 'Physical Exercise*'[Title/Abstract] OR 'Physical Activit*'[Title/Abstract] OR 'Aerobic Exercise*'[Title/Abstract] OR 'Isometric Exercise*'[Title/Abstract] OR 'Acute Exercise*'[Title/Abstract] OR 'Exercise Training*'[Title/Abstract] OR 'biometric exercise'[Title/Abstract] OR 'effort'[Title/Abstract] OR 'exercise capacity'[Title/Abstract] OR 'exercise performance'[Title/Abstract] OR 'exertion'[Title/Abstract] OR 'fitness training'[Title/Abstract] OR 'fitness workout'[Title/Abstract] OR 'physical effort'[Title/Abstract] OR 'physical exertion'[Title/Abstract] OR 'physical workout'[Title/Abstract] OR 'exercise'[Title/Abstract] |
| #6 | ("Exercise"[Mesh]) OR ('Exercises'[Title/Abstract] OR 'Physical Exercise*'[Title/Abstract] OR 'Physical Activit*'[Title/Abstract] OR 'Aerobic Exercise*'[Title/Abstract] OR 'Isometric Exercise*'[Title/Abstract] OR 'Acute Exercise*'[Title/Abstract] OR 'Exercise Training*'[Title/Abstract] OR 'biometric exercise'[Title/Abstract] OR 'effort'[Title/Abstract] OR 'exercise capacity'[Title/Abstract] OR 'exercise performance'[Title/Abstract] OR 'exertion'[Title/Abstract] OR 'fitness training'[Title/Abstract] OR 'fitness workout'[Title/Abstract] OR 'physical effort'[Title/Abstract] OR 'physical exertion'[Title/Abstract] OR 'physical workout'[Title/Abstract] OR 'exercise'[Title/Abstract]) |
| #7 | "Resistance Training"[Mesh] |
| #8 | 'Strength Training'[Title/Abstract] OR 'Weight Lifting Strengthening Program*'[Title/Abstract] OR 'Weight Lifting Exercise Program*'[Title/Abstract] OR 'Weight Bearing Strengthening Program*'[Title/Abstract] OR 'Weight Bearing Exercise Program*'[Title/Abstract] OR 'resistance exercise'[Title/Abstract] OR 'resistance exercise training'[Title/Abstract] OR 'resistance-type exercise'[Title/Abstract] OR 'resistance-type training'[Title/Abstract] OR 'strength-type exercise'[Title/Abstract] OR 'strength-type training'[Title/Abstract] OR 'resistance training'[Title/Abstract] |
| #9 | ("Resistance Training"[Mesh]) OR ('Strength Training'[Title/Abstract] OR 'Weight Lifting Strengthening Program*'[Title/Abstract] OR 'Weight Lifting Exercise Program*'[Title/Abstract] OR 'Weight Bearing Strengthening Program*'[Title/Abstract] OR 'Weight Bearing Exercise Program*'[Title/Abstract] OR 'resistance exercise'[Title/Abstract] OR 'resistance exercise training'[Title/Abstract] OR 'resistance-type exercise'[Title/Abstract] OR 'resistance-type training'[Title/Abstract] OR 'strength-type exercise'[Title/Abstract] OR 'strength-type training'[Title/Abstract] OR 'resistance training'[Title/Abstract]) |
| #10 | "High-Intensity Interval Training"[Mesh] |
| #11 | 'High Intensity Interval Training*'[Title/Abstract] OR 'High-Intensity Intermittent Exercise*'[Title/Abstract] OR 'Sprint Interval Training*'[Title/Abstract] OR 'high-intensity intermittent training'[Title/Abstract] OR 'high-intensity interval exercise'[Title/Abstract] OR 'high-intensity interval training'[Title/Abstract] OR 'HIIE'[Title/Abstract] OR 'HIIT'[Title/Abstract] OR 'intermittent high-intensity training'[Title/Abstract] OR 'interval high-intensity training'[Title/Abstract] |
| #12 | ("High-Intensity Interval Training"[Mesh]) OR ('High Intensity Interval Training*'[Title/Abstract] OR 'High-Intensity Intermittent Exercise*'[Title/Abstract] OR 'Sprint Interval Training*'[Title/Abstract] OR 'high-intensity intermittent training'[Title/Abstract] OR 'high-intensity interval exercise'[Title/Abstract] OR 'high-intensity interval training'[Title/Abstract] OR 'HIIE'[Title/Abstract] OR 'HIIT'[Title/Abstract] OR 'intermittent high-intensity training'[Title/Abstract] OR 'interval high-intensity training'[Title/Abstract]) |
| #13 | ((("Exercise"[Mesh]) OR ('Exercises'[Title/Abstract] OR 'Physical Exercise*'[Title/Abstract] OR 'Physical Activit*'[Title/Abstract] OR 'Aerobic Exercise*'[Title/Abstract] OR 'Isometric Exercise*'[Title/Abstract] OR 'Acute Exercise*'[Title/Abstract] OR 'Exercise Training*'[Title/Abstract] OR 'biometric exercise'[Title/Abstract] OR 'effort'[Title/Abstract] OR 'exercise capacity'[Title/Abstract] OR 'exercise performance'[Title/Abstract] OR 'exertion'[Title/Abstract] OR 'fitness training'[Title/Abstract] OR 'fitness workout'[Title/Abstract] OR 'physical effort'[Title/Abstract] OR 'physical exertion'[Title/Abstract] OR 'physical workout'[Title/Abstract] OR 'exercise'[Title/Abstract])) OR (("Resistance Training"[Mesh]) OR ('Strength Training'[Title/Abstract] OR 'Weight Lifting Strengthening Program*'[Title/Abstract] OR 'Weight Lifting Exercise Program*'[Title/Abstract] OR 'Weight Bearing Strengthening Program*'[Title/Abstract] OR 'Weight Bearing Exercise Program*'[Title/Abstract] OR 'resistance exercise'[Title/Abstract] OR 'resistance exercise training'[Title/Abstract] OR 'resistance-type exercise'[Title/Abstract] OR 'resistance-type training'[Title/Abstract] OR 'strength-type exercise'[Title/Abstract] OR 'strength-type training'[Title/Abstract] OR 'resistance training'[Title/Abstract]))) OR (("High-Intensity Interval Training"[Mesh]) OR ('High Intensity Interval Training*'[Title/Abstract] OR 'High-Intensity Intermittent Exercise*'[Title/Abstract] OR 'Sprint Interval Training*'[Title/Abstract] OR 'high-intensity intermittent training'[Title/Abstract] OR 'high-intensity interval exercise'[Title/Abstract] OR 'high-intensity interval training'[Title/Abstract] OR 'HIIE'[Title/Abstract] OR 'HIIT'[Title/Abstract] OR 'intermittent high-intensity training'[Title/Abstract] OR 'interval high-intensity training'[Title/Abstract])) |
| #14 | (("Stroke"[Mesh]) OR ('Strokes'[Title/Abstract] OR 'Cerebrovascular Accident*'[Title/Abstract] OR 'Cerebral Stroke*'[Title/Abstract] OR 'Cerebrovascular Apoplexy'[Title/Abstract] OR 'Brain Vascular Accident*'[Title/Abstract] OR 'Cerebrovascular Stroke*'[Title/Abstract] OR 'Apoplexy'[Title/Abstract] OR 'CVA'[Title/Abstract] OR 'CVAs'[Title/Abstract] OR 'Cerebrovascular Accident'[Title/Abstract] OR 'Acute Stroke*'[Title/Abstract] OR 'Acute Cerebrovascular Accident*'[Title/Abstract] OR 'acute cerebrovascular lesion'[Title/Abstract] OR 'acute focal cerebral vasculopathy'[Title/Abstract] OR 'apoplectic stroke'[Title/Abstract] OR 'apoplexia'[Title/Abstract] OR 'brain accident'[Title/Abstract] OR 'brain attack'[Title/Abstract] OR 'brain blood flow disturbance'[Title/Abstract] OR 'brain insult'[Title/Abstract] OR 'brain insultus'[Title/Abstract] OR 'cerebral apoplexia'[Title/Abstract] OR 'cerebral insult'[Title/Abstract] OR 'cerebral vascular accident'[Title/Abstract] OR 'cerebral vascular insufficiency'[Title/Abstract] OR 'cerebro vascular accident'[Title/Abstract] OR 'cerebrovascular arrest'[Title/Abstract] OR 'cerebrovascular failure'[Title/Abstract] OR 'cerebrovascular injury'[Title/Abstract] OR 'cerebrovascular insufficiency'[Title/Abstract] OR 'cerebrovascular insult'[Title/Abstract] OR 'cerebrum vascular accident'[Title/Abstract] OR 'cryptogenic stroke'[Title/Abstract] OR 'insultus cerebralis'[Title/Abstract] OR 'ischaemic seizure'[Title/Abstract] OR 'ischemic seizure'[Title/Abstract] OR 'stroke'[Title/Abstract] OR 'thrombotic stroke'[Title/Abstract])) AND (((("Exercise"[Mesh]) OR ('Exercises'[Title/Abstract] OR 'Physical Exercise*'[Title/Abstract] OR 'Physical Activit*'[Title/Abstract] OR 'Aerobic Exercise*'[Title/Abstract] OR 'Isometric Exercise*'[Title/Abstract] OR 'Acute Exercise*'[Title/Abstract] OR 'Exercise Training*'[Title/Abstract] OR 'biometric exercise'[Title/Abstract] OR 'effort'[Title/Abstract] OR 'exercise capacity'[Title/Abstract] OR 'exercise performance'[Title/Abstract] OR 'exertion'[Title/Abstract] OR 'fitness training'[Title/Abstract] OR 'fitness workout'[Title/Abstract] OR 'physical effort'[Title/Abstract] OR 'physical exertion'[Title/Abstract] OR 'physical workout'[Title/Abstract] OR 'exercise'[Title/Abstract])) OR (("Resistance Training"[Mesh]) OR ('Strength Training'[Title/Abstract] OR 'Weight Lifting Strengthening Program*'[Title/Abstract] OR 'Weight Lifting Exercise Program*'[Title/Abstract] OR 'Weight Bearing Strengthening Program*'[Title/Abstract] OR 'Weight Bearing Exercise Program*'[Title/Abstract] OR 'resistance exercise'[Title/Abstract] OR 'resistance exercise training'[Title/Abstract] OR 'resistance-type exercise'[Title/Abstract] OR 'resistance-type training'[Title/Abstract] OR 'strength-type exercise'[Title/Abstract] OR 'strength-type training'[Title/Abstract] OR 'resistance training'[Title/Abstract]))) OR (("High-Intensity Interval Training"[Mesh]) OR ('High Intensity Interval Training*'[Title/Abstract] OR 'High-Intensity Intermittent Exercise*'[Title/Abstract] OR 'Sprint Interval Training*'[Title/Abstract] OR 'high-intensity intermittent training'[Title/Abstract] OR 'high-intensity interval exercise'[Title/Abstract] OR 'high-intensity interval training'[Title/Abstract] OR 'HIIE'[Title/Abstract] OR 'HIIT'[Title/Abstract] OR 'intermittent high-intensity training'[Title/Abstract] OR 'interval high-intensity training'[Title/Abstract]))) |
| #15 | "Randomized Controlled Trial" [Publication Type] |
| #16 | 'controlled trial, randomized'[Title/Abstract] OR 'randomised controlled study'[Title/Abstract] OR 'randomised controlled trial'[Title/Abstract] OR 'randomized controlled study'[Title/Abstract] OR 'randomized controlled trial'[Title/Abstract] OR 'rct'[Title/Abstract] OR 'RCT'[Title/Abstract] |
| #17 | ("Randomized Controlled Trial" [Publication Type]) OR ('controlled trial, randomized'[Title/Abstract] OR 'randomised controlled study'[Title/Abstract] OR 'randomised controlled trial'[Title/Abstract] OR 'randomized controlled study'[Title/Abstract] OR 'randomized controlled trial'[Title/Abstract] OR 'rct'[Title/Abstract] OR 'RCT'[Title/Abstract]) |
| #18 | ((("Stroke"[Mesh]) OR ('Strokes'[Title/Abstract] OR 'Cerebrovascular Accident*'[Title/Abstract] OR 'Cerebral Stroke*'[Title/Abstract] OR 'Cerebrovascular Apoplexy'[Title/Abstract] OR 'Brain Vascular Accident*'[Title/Abstract] OR 'Cerebrovascular Stroke*'[Title/Abstract] OR 'Apoplexy'[Title/Abstract] OR 'CVA'[Title/Abstract] OR 'CVAs'[Title/Abstract] OR 'Cerebrovascular Accident'[Title/Abstract] OR 'Acute Stroke*'[Title/Abstract] OR 'Acute Cerebrovascular Accident*'[Title/Abstract] OR 'acute cerebrovascular lesion'[Title/Abstract] OR 'acute focal cerebral vasculopathy'[Title/Abstract] OR 'apoplectic stroke'[Title/Abstract] OR 'apoplexia'[Title/Abstract] OR 'brain accident'[Title/Abstract] OR 'brain attack'[Title/Abstract] OR 'brain blood flow disturbance'[Title/Abstract] OR 'brain insult'[Title/Abstract] OR 'brain insultus'[Title/Abstract] OR 'cerebral apoplexia'[Title/Abstract] OR 'cerebral insult'[Title/Abstract] OR 'cerebral vascular accident'[Title/Abstract] OR 'cerebral vascular insufficiency'[Title/Abstract] OR 'cerebro vascular accident'[Title/Abstract] OR 'cerebrovascular arrest'[Title/Abstract] OR 'cerebrovascular failure'[Title/Abstract] OR 'cerebrovascular injury'[Title/Abstract] OR 'cerebrovascular insufficiency'[Title/Abstract] OR 'cerebrovascular insult'[Title/Abstract] OR 'cerebrum vascular accident'[Title/Abstract] OR 'cryptogenic stroke'[Title/Abstract] OR 'insultus cerebralis'[Title/Abstract] OR 'ischaemic seizure'[Title/Abstract] OR 'ischemic seizure'[Title/Abstract] OR 'stroke'[Title/Abstract] OR 'thrombotic stroke'[Title/Abstract])) AND (((("Exercise"[Mesh]) OR ('Exercises'[Title/Abstract] OR 'Physical Exercise*'[Title/Abstract] OR 'Physical Activit*'[Title/Abstract] OR 'Aerobic Exercise*'[Title/Abstract] OR 'Isometric Exercise*'[Title/Abstract] OR 'Acute Exercise*'[Title/Abstract] OR 'Exercise Training*'[Title/Abstract] OR 'biometric exercise'[Title/Abstract] OR 'effort'[Title/Abstract] OR 'exercise capacity'[Title/Abstract] OR 'exercise performance'[Title/Abstract] OR 'exertion'[Title/Abstract] OR 'fitness training'[Title/Abstract] OR 'fitness workout'[Title/Abstract] OR 'physical effort'[Title/Abstract] OR 'physical exertion'[Title/Abstract] OR 'physical workout'[Title/Abstract] OR 'exercise'[Title/Abstract])) OR (("Resistance Training"[Mesh]) OR ('Strength Training'[Title/Abstract] OR 'Weight Lifting Strengthening Program*'[Title/Abstract] OR 'Weight Lifting Exercise Program*'[Title/Abstract] OR 'Weight Bearing Strengthening Program*'[Title/Abstract] OR 'Weight Bearing Exercise Program*'[Title/Abstract] OR 'resistance exercise'[Title/Abstract] OR 'resistance exercise training'[Title/Abstract] OR 'resistance-type exercise'[Title/Abstract] OR 'resistance-type training'[Title/Abstract] OR 'strength-type exercise'[Title/Abstract] OR 'strength-type training'[Title/Abstract] OR 'resistance training'[Title/Abstract]))) OR (("High-Intensity Interval Training"[Mesh]) OR ('High Intensity Interval Training*'[Title/Abstract] OR 'High-Intensity Intermittent Exercise*'[Title/Abstract] OR 'Sprint Interval Training*'[Title/Abstract] OR 'high-intensity intermittent training'[Title/Abstract] OR 'high-intensity interval exercise'[Title/Abstract] OR 'high-intensity interval training'[Title/Abstract] OR 'HIIE'[Title/Abstract] OR 'HIIT'[Title/Abstract] OR 'intermittent high-intensity training'[Title/Abstract] OR 'interval high-intensity training'[Title/Abstract])))) AND (("Randomized Controlled Trial" [Publication Type]) OR ('controlled trial, randomized'[Title/Abstract] OR 'randomised controlled study'[Title/Abstract] OR 'randomised controlled trial'[Title/Abstract] OR 'randomized controlled study'[Title/Abstract] OR 'randomized controlled trial'[Title/Abstract] OR 'rct'[Title/Abstract] OR 'RCT'[Title/Abstract])) |

S1.4 Search Strategies for Cochrane Library

| NO. | Query |
| --- | --- |
| #1 | MeSH descriptor: [Stroke] explode all trees |
| #2 | ('Strokes' OR 'Cerebrovascular Accident*' OR 'Cerebral Stroke*' OR 'Cerebrovascular Apoplexy' OR 'Brain Vascular Accident*' OR 'Cerebrovascular Stroke*' OR 'Apoplexy' OR 'CVA' OR 'CVAs' OR 'Cerebrovascular Accident' OR 'Acute Stroke*' OR 'Acute Cerebrovascular Accident*' OR 'acute cerebrovascular lesion' OR 'acute focal cerebral vasculopathy' OR 'apoplectic stroke' OR 'apoplexia' OR 'brain accident' OR 'brain attack' OR 'brain blood flow disturbance' OR 'brain insult' OR 'brain insultus' OR 'cerebral apoplexia' OR 'cerebral insult' OR 'cerebral vascular accident' OR 'cerebral vascular insufficiency' OR 'cerebro vascular accident' OR 'cerebrovascular arrest' OR 'cerebrovascular failure' OR 'cerebrovascular injury' OR 'cerebrovascular insufficiency' OR 'cerebrovascular insult' OR 'cerebrum vascular accident' OR 'cryptogenic stroke' OR 'insultus cerebralis' OR 'ischaemic seizure' OR 'ischemic seizure' OR 'stroke' OR 'thrombotic stroke'):ti,ab,kw (Word variations have been searched) |
| #3 | #1 OR #2 |
| #4 | MeSH descriptor: [Exercise] explode all trees |
| #5 | ('Exercises' OR 'Physical Exercise*' OR 'Physical Activity*' OR 'Aerobic Exercise*' OR 'Isometric Exercise*' OR 'Acute Exercise*' OR 'Exercise Training*' OR 'biometric exercise' OR 'effort' OR 'exercise capacity' OR 'exercise performance' OR 'exertion' OR 'fitness training' OR 'fitness workout' OR 'physical effort' OR 'physical exertion' OR 'physical workout' OR 'exercise'):ti,ab,kw (Word variations have been searched) |
| #6 | #4 OR #5 |
| #7 | MeSH descriptor: [Resistance Training] explode all trees |
| #8 | ('Strength Training' OR 'Weight Lifting Strengthening Program*' OR 'Weight Lifting Exercise Program*' OR 'Weight Bearing Strengthening Program*' OR 'Weight Bearing Exercise Program*' OR 'resistance exercise' OR 'resistance exercise training' OR 'resistance-type exercise' OR 'resistance-type training' OR 'strength-type exercise' OR 'strength-type training' OR 'resistance training'):ti,ab,kw (Word variations have been searched) |
| #9 | #7 OR #8 |
| #10 | MeSH descriptor: [High-Intensity Interval Training] explode all trees |
| #11 | ('High Intensity Interval Training*' OR 'High-Intensity Intermittent Exercise*' OR 'Sprint Interval Training*' OR 'high-intensity intermittent training' OR 'high-intensity interval exercise' OR 'high-intensity interval training' OR 'HIIE' OR 'HIIT' OR 'intermittent high-intensity training' OR 'interval high-intensity training'):ti,ab,kw (Word variations have been searched) |
| #12 | #10 OR #11 |
| #13 | #6 OR #9 OR #12 |
| #14 | #3 AND #13 |
| #15 | MeSH descriptor: [Randomized Controlled Trial] explode all trees |
| #16 | ('controlled trial, randomized' OR 'randomised controlled study' OR 'randomised controlled trial' OR 'randomized controlled study' OR 'randomized controlled trial' OR 'rct' OR 'RCT'):ti,ab,kw (Word variations have been searched) |
| #17 | #15 OR #16 |
| #18 | #14 AND #17 |

| **Risk indicator** | **β** | **95%CI** | **I^2^** |
| --- | --- | --- | --- |
| **AE v CT** | | | |
| 年龄 | 1.842 | (-2.156 to 5.840) | 12% |
| 干预时长 | 3.927 | (0.891 to 6.963) | 9% |
| **RE v CT** | | | |
| 年龄 | 2.156 | (-1.843 to 6.155) | 13% |
| 干预时长 | 2.834 | (-0.532 to 6.200) | 10% |
| **AE_RE v AE** | | | |
| 年龄 | -0.784 | (-4.236 to 2.668) | 11% |
| 干预时长 | 1.523 | (-1.845 to 4.891) | 10% |
| **ME v CT** | | | |
| 年龄 | 1.325 | (-3.127 to 5.777) | 14% |
| 干预时长 | 4.215 | (1.028 to 7.402) | 8% |

**Table S2. A)** Meta regression analysis: The influence of age and intervention duration on 6MWD.

**Table S2. B)** Meta regression analysis: The influence of age and intervention duration on 10MWT.

| **Risk indicator** | **β** | **95%CI** | **I^2^** |
| --- | --- | --- | --- |
| **AE v CT** | | | |
| 年龄 | -0.824 | (-2.156 to 0.508) | 15% |
| 干预时长 | 1.927 | (0.491 to 3.363) | 12% |
| **AE_RE v AE** | | | |
| 年龄 | 0.456 | (-1.236 to 2.148) | 13% |
| 干预时长 | 0.823 | (-0.845 to 2.491) | 11% |
| **CSE v CT** | | | |
| 年龄 | -1.125 | (-3.127 to 0.877) | 16% |
| 干预时长 | 2.415 | (0.728 to 4.102) | 11% |
| **ME v CT** |  |  |  |
| 年龄 | -0.725 | (-2.627 to 1.177) | 14% |
| 干预时长 | 1.615 | (0.228 to 3.002) | 10% |
| **PME v CT** | | | |
| 年龄 | 0.325 | (-1.427 to 2.077) | 15% |
| 干预时长 | 0.915 | (-0.532 to 2.362) | 12% |

**Table S2. C)** Meta regression analysis: The influence of age and intervention duration on BI.

| **Risk indicator** | **β** | **95%CI** | **I²** |
| --- | --- | --- | --- |
| **AE v CT** | | | |
| 年龄 | 1.842 | (-2.156 to 5.840) | 12% |
| 干预时长 | 3.927 | (0.891 to 6.963) | 9% |
| **RE v CT** | | | |
| 年龄 | 2.156 | (-1.843 to 6.155) | 13% |
| 干预时长 | 2.834 | (-0.532 to 6.200) | 10% |
| **CSE v CT** | | | |
| 年龄 | -1.125 | (-3.127 to 0.877) | 16% |
| 干预时长 | 2.415 | (0.728 to 4.102) | 11% |
| **ME v CT** | | | |
| 年龄 | -0.725 | (-2.627 to 1.177) | 14% |
| 干预时长 | 1.615 | (0.228 to 3.002) | 10% |
| **PME v CT** | | | |
| 年龄 | 0.325 | (-1.427 to 2.077) | 15% |
| 干预时长 | 0.915 | (-0.532 to 2.362) | 12% |

**Table S2. D)** Meta regression analysis: The influence of age and intervention duration on FMA.

| **Risk indicator** | **β** | **95%CI** | **I²** |
| --- | --- | --- | --- |
| **AE v CT** | | | |
| 年龄 | -0.824 | (-2.156 to 0.508) | 15% |
| 干预时长 | 1.927 | (0.491 to 3.363) | 12% |
| **AE_RE v AE** | | | |
| 年龄 | 0.456 | (-1.236 to 2.148) | 13% |
| 干预时长 | 0.823 | (-0.845 to 2.491) | 11% |
| **CSE v CT** | | | |
| 年龄 | -1.125 | (-3.127 to 0.877) | 16% |
| 干预时长 | 2.415 | (0.728 to 4.102) | 11% |
| **ME v CT** | | | |
| 年龄 | -0.725 | (-2.627 to 1.177) | 14% |
| 干预时长 | 1.615 | (0.228 to 3.002) | 10% |
| **PME v CT** | | | |
| 年龄 | 0.325 | (-1.427 to 2.077) | 15% |
| 干预时长 | 0.915 | (-0.532 to 2.362) | 12% |

**Table S3. A)** Assessment of the Confidence in the Evidence: The GRADE on 6MWD.


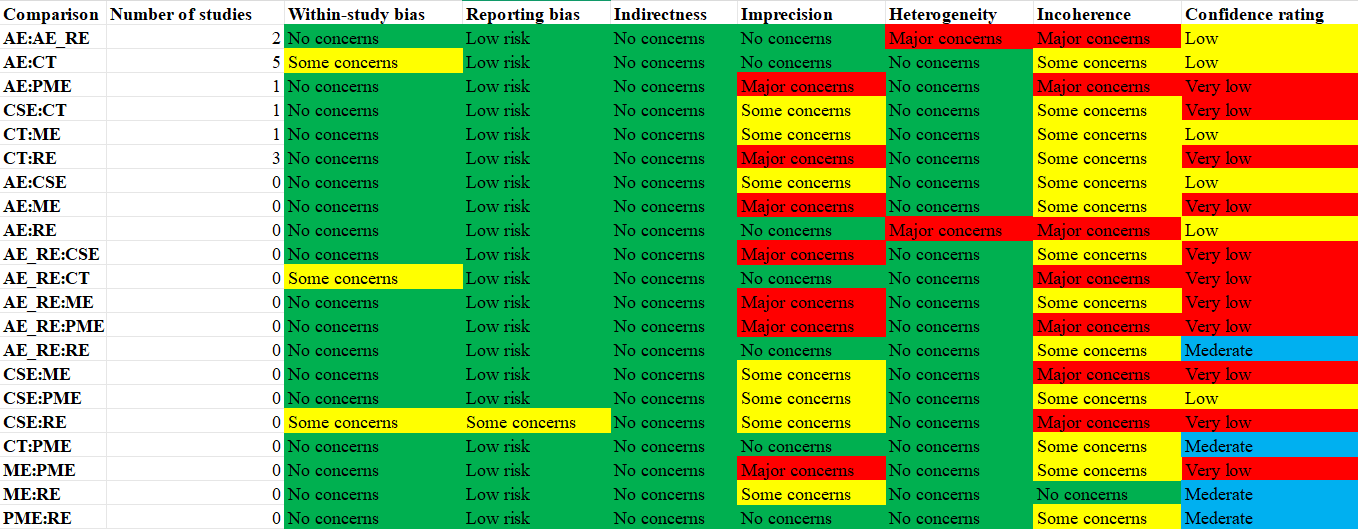


**Table S3. B)** Assessment of the Confidence in the Evidence: The GRADE on 10MWT.


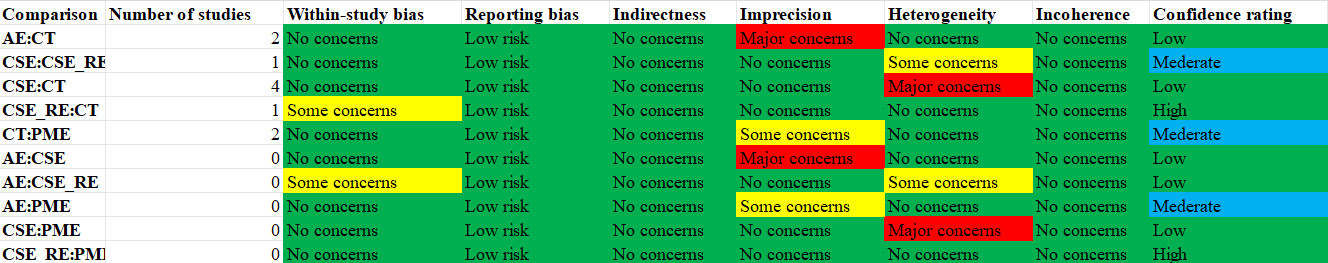


**Table S3. C)** Assessment of the Confidence in the Evidence: The GRADE on BI.


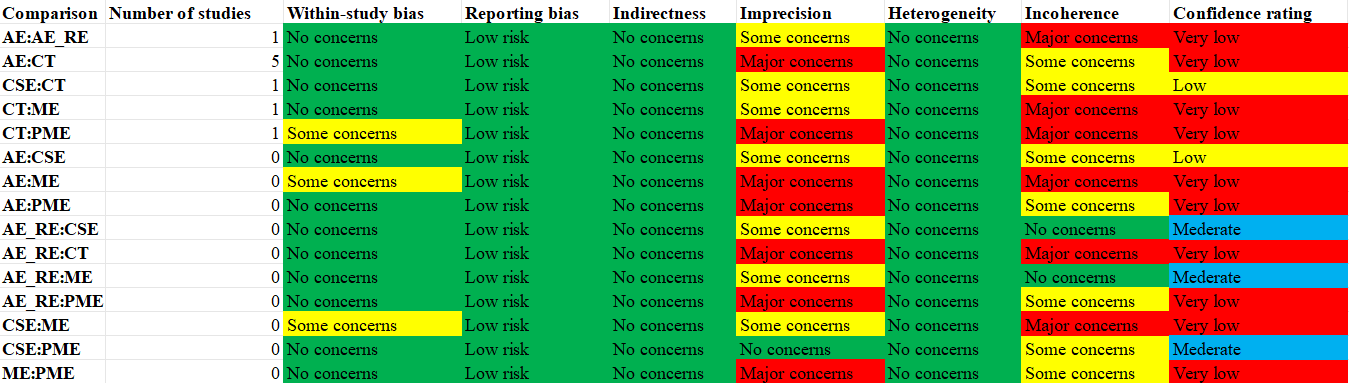


**Table S3. D)** Assessment of the Confidence in the Evidence: The GRADE on FMA.


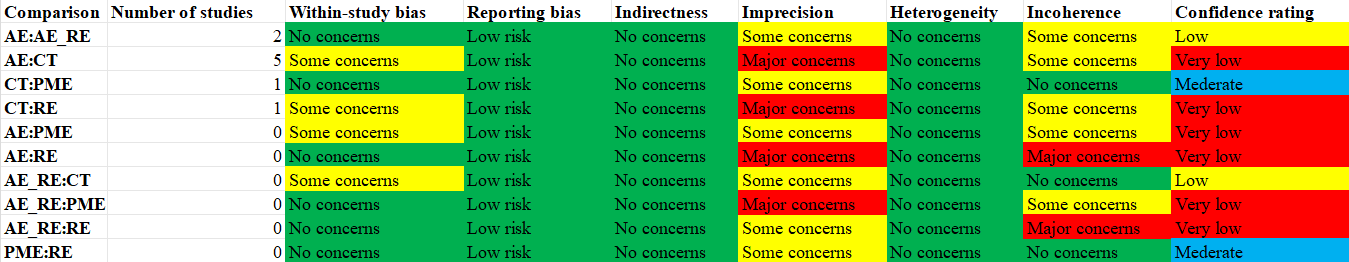

Supplement: Supplementary file 1 [file Table_1.DOCX]
